# Embryonic and post-embryonic development of the polyclad flatworm *Maritigrella crozieri*; implications for the evolution of spiralian life history traits

**DOI:** 10.1186/1742-9994-7-12

**Published:** 2010-04-28

**Authors:** Kate A Rawlinson

**Affiliations:** 1 Smithsonian Marine Station, 701 Seaway Drive, Fort Pierce, Florida, 34949 USA; 2 Current address: Department of Genetics, Evolution and Environment, University College London, Gower Street, London WC1E 6BT, UK

## Abstract

**Background:**

Planktonic life history stages of spiralians share some muscular, nervous and ciliary system characters in common. The distribution of these characters is patchy and can be interpreted either as the result of convergent evolution, or as the retention of primitive spiralian larval features. To understand the evolution of these characters adequate taxon sampling across the Spiralia is necessary. Polyclad flatworms are the only free-living Platyhelminthes that exhibit a continuum of developmental modes, with direct development at one extreme, and indirect development via a trochophore-like larval stage at the other. Here I present embryological and larval anatomical data from the indirect developing polyclad *Maritrigrella crozieri*, and consider these data within a comparative spiralian context.

**Results:**

After 196 h hours of embryonic development, *M. crozieri *hatches as a swimming, planktotrophic larva. Larval myoanatomy consists of an orthogonal grid of circular and longitudinal body wall muscles plus parenchymal muscles. Diagonal body wall muscles develop over the planktonic period. Larval neuroanatomy consists of an apical plate, neuropile, paired nerve cords, a peri-oral nerve ring, a medial nerve, a ciliary band nerve net and putative ciliary photoreceptors. Apical neural elements develop first followed by posterior perikarya and later pharyngeal neural elements. The ciliated larva is encircled by a continuous, pre-oral band of longer cilia, which follows the distal margins of the lobes; it also possesses distinct apical and caudal cilia.

**Conclusions:**

Within polyclads heterochronic shifts in the development of diagonal bodywall and pharyngeal muscles are correlated with life history strategies and feeding requirements. In contrast to many spiralians, *M. crozieri *hatch with well developed nervous and muscular systems. Comparisons of the ciliary bands and apical organs amongst spiralian planktonic life-stages reveal differences; *M. crozieri *lack a distinct ciliary band muscle and flask-shaped epidermal serotonergic cells of the apical organ. Based on current phylogenies, the distribution of ciliary bands and apical organs between polyclads and other spiralians is not congruent with a hypothesis of homology. However, some similarities exist, and this study sets an anatomical framework from which to investigate cellular and molecular mechanisms that will help to distinguish between parallelism, convergence and homology of these features.

## Background

Flatworms (phylum Platyhelminthes) have long been viewed as the sister group to all other bilaterian metazoans, due in large part to their blind gut and relatively simple, acoelomate body plan [1]. However, recent molecular phylogenetic studies have forced a re-evaluation of classical hypotheses of metazoan intrarelationships. The Platyhelminthes (including catenulids and rhabditophorans, but excluding the acoels and nemertodermatids [2]) now nest within the Spiralia, the protostomian sister clade to Ecdysozoa [[3] based on [4]]. Resolution of intrarelationships within the Spiralia is increasing, and the Platyhelminths are thought to belong to a clade that includes the Bryozoa, Entoprocta, Cycliophora [5], and possibly Gastrotricha and Gnathostomulida [6]. This clade, in turn, is the sister group to a clade that includes annelids, mollusks, nemerteans and brachiopods [4,6]. This new phylogenic hypothesis has a number of profound implications for scenarios of metazoan morphological and life history evolution. This is largely due to the patchy distribution of ciliated planktonic ("larval") stages across spiralians and deuterostomes.

A cursory examination of the phylogenetic distribution of larval stages within the mollusk/annelid/nemertean/branchiopod clade suggests that a ciliated "trochophore" larva could have been a primitive feature of this group. However, within its sister clade (Platyhelminthes, Gastrotricha, Gnathostomulida, Bryozoa, Entoprocta, Cycliophora) the distribution of larval forms is scarcer, and absent in the gastrotrichs and gnathostomulids. This distribution of larval characters within the Spiralia could be interpreted in two ways: either as the result of repeated convergent evolution of intermediate planktonic life stages [7], or, perhaps more controversially, as the retention of a primitive spiralian larval form in certain groups, with multiple instances of independent loss in other lineages [8]. To distinguish between these alternative hypotheses of character evolution, detailed morphological and developmental data are needed from a wide diversity of taxa within the Spiralia - and in particular, from the relatively understudied indirect developing members of the Platyhelminthes, Bryozoa, Entoprocta, Cycliophora. Polyclad flatworms are unique among the free-living marine platyhelminths in having a gradient of developmental modes. These modes of development can be roughly categorized as direct development (embryos hatching as a benthic juvenile), intermediate development (larva with lobes and ciliary band retained within an egg case, and hatching as a benthic juvenile), and indirect development (with a planktonic form with lobes and ciliary band) [9]. Polyclad planktonic larval forms are, themselves, morphologically diverse, and include the four-lobed Götte's larva, the eight-lobed Müller's larva, as well as additional six- and ten-lobed forms [[10,11], pers obs]. To date, Müller's larvae have been described in both suborders of polyclads - the Cotylea and Acotylea - while Götte's larva, intracapsular larva and direct development are all found exclusively with the Acotylea [12]. However, a dearth of data on the intrarelationships of polyclads and insufficient taxonomic sampling of their life history strategies precludes inference of the primitive mode of development for this clade.

The ciliated larvae of polyclads have been thought to superficially resemble the pilidium of nemertines [10], and it has been suggested - based on morphological and topological similarities between their ciliary bands and cephalic ganglia - that polyclad larvae are homologous with the trochophore larva of other spiralians [13]. However, current hypotheses of platyhelminth phylogeny support direct development as the primitive condition for the clade [2,14,15], and the appearance of ciliated larval forms within a single order of free-living platyhelminths may, instead, point to the convergent evolution of a planktonic life stage in polyclads. Nevertheless, due to the variable position of the Platyhelminthes within the Spiralia [e.g. [5] and [6]] and of the Polycladida within the Platyhelminthes [see [16]] the question of polyclad-trochophore larval homology or convergence remains open, and comparative analyses of the development of putatively homologous larval structures are needed to distinguish between these alternative scenarios.

Larval characters that are widespread amongst spiralian larvae include an apical tuft, paired ventral nerve cords, paired lateral nerve cords, cerebral ganglia, circumoral nerve loops, perikarya associated with the pedal nerve cord and the establishment of an apical muscle grid during myogenesis [17,18]. To date, descriptive studies on indirect developing polyclads using modern microscopy and fluorescent labeling techniques have characterized two anatomical similarities shared between spiralian and polyclad larvae - the early establishment of an apical muscle grid in the acotylean *Hoploplana inquilina *[19] and cotylean *Maritigrella crozieri *[20], and paired ventral and lateral nerve cords in the acotylean *Imogine mcgrathi *[21]. Ultrastructural studies of polyclad larvae have greatly increased our understanding of the peripheral and central nervous system (including the brain and ciliary band - [21-24]). However, many aspects of polyclad embryonic and post-embryonic development remain unknown. For instance, the fate of the primary longitudinal muscles and development of pharyngeal muscles is undescribed, and data on the development and distribution of embryonic and larval neurons, their spatial relationship to the embryonic and larval musculature, and the development of the ciliary band and apical organs are lacking.

Here, I provide a detailed account of embryonic and larval development in the cotylean polyclad *Maritigrella crozieri*. Using a nuclear stain, phalloidin staining for F-actin, and immunohistochemical staining of the nervous system, I show in detail: early cleavage, gastrulation and development of the ciliary band; myogenesis and neurogenesis. I discuss how different temporal patterns of bodywall muscle development relate to different life history strategies within polyclads. This first analysis of neurotransmittor distribution shows a well-developed nervous system in the hatchling of *M. crozieri*. Myogenic and neurogenic elements of the ciliary band and apical plate in *M. crozieri *are compared to those in other spiralian pelagic life history stages. Evidence for and against homology of these organs is given, however, these data are not sufficient to make the non-arbitrary distinction between homology, parallelism and convergence. Efforts needed to decipher between these three scenarios are discussed and this study provides a morphogenetic and embryological foundation in polyclads for future comparative studies of spiralian developmental gene expression and function.

## Results

### Overview of *Maritigrella crozieri *embryogenesis

In a quiescent state mature *Maritigrella crozieri *measure 31.3 (± 2.7) mm in length and 18.1 (± 1.9) mm in width (mean ± SD; *n *= 20). Individuals lay multiple egg batches, each containing anywhere from 50 to over 1000 individually encapsulated eggs. The eggs are nearly spherical and measure *220 (± 15.6) *μm (mean ± SD, *n *= 20) in diameter. At 22°C embryonic development took 196 (± 16) h (mean + SD, *n *= 200) to hatching. Embryonic stages are described as hours post oviposition (hpo) and, in order to make them comparable between taxa, as a percentage of embryonic development time (% d.t.). There was no difference in development time between encapsulated embryos and those reared outside of the egg capsule.

**Figure 1:**
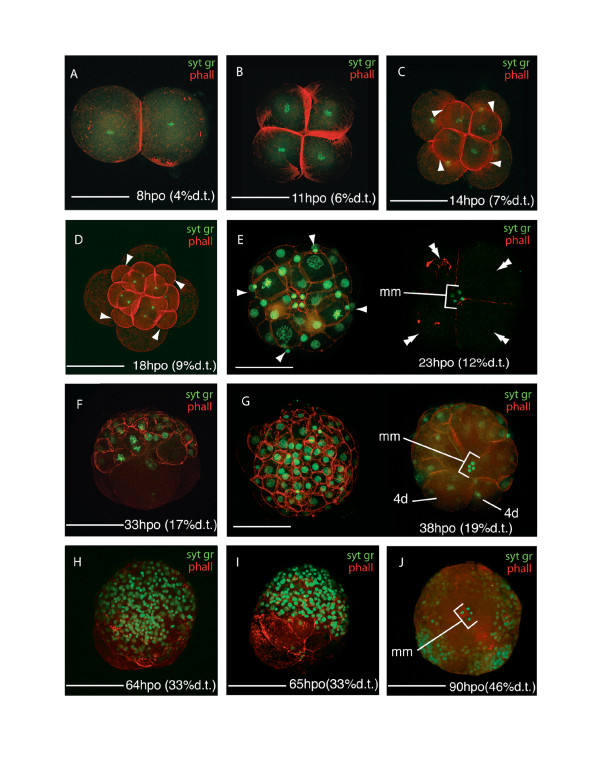
**Confocal laser scanning micrographs (CLSM) of the blastula and gastrula stages of *Maritigrella crozieri***. (F-actin labeled with phalloidin 564 [red], nucleic acids stained with Sytox Green [green]). **(A-D) **- animal views of cleavage cycles 1-4; showing the first quartet micromeres (arrowheads in C) and second quartet micromeres (arrowheads in D). **(E) **Animal view showing the third quartet micromeres (arrowheads), vegetal view showing large fourth quartet micromeres (4a-d, double arrowheads) and mini-macromeres (*mm*)(4A-D). **(F) **Lateral view shows irregular double layer of dividing 1^st ^to 3^rd ^quartet micromeres at the animal pole over the 4a-d quartet. **(G) **Animal view of micromere cap and vegetal view of 4d division. **(H-J) **Animal, lateral and vegetal views respectively of gastrulation by epiboly; micromere cap migrates vegetally enveloping the fourth quartet micromeres and macromeres, which remain visible until the ectoderm invaginates at the lower pole to form the stomodeum. Scale bars 100 μm, hpo = hours post oviposition, dt = development time.

Cleavage in *Maritigrella crozieri *is quartet, spiral and equal. The first cleavage takes place 8 hpo (4% d.t., Fig. 1A), followed by the second cleavage at 11 hpo (6% d.t., Fig. 1B). The four blastomeres are the same size making quadrant identification difficult. Following the cleavage nomenclature of Surface [25], the first quartet micromeres develop at 14 hpo (7% d.t., Fig. 1C), the second quartet micromeres at 18 hpo (9% d.t., Fig. 1D), the very small third quartet micromeres at 21 hpo (11% d.t.), and the large fourth quartet micromeres (~90 μm) and very small macromeres (10 μm) at 23 hpo (12% d.t., Fig. 1E). The number of micromeres from the first three quartets increases to more than 83 in the next 10 hours, these form an irregular double layer at the animal pole and form a cap over the 4th quartet micromeres (Fig. 1F). At the onset of gastrulation a 4^th ^quartet micromere divides (33 hpo, 17% d.t., Fig. 1G) - this, according to Surface [25], is 4d. Gastrulation proceeds by epiboly - the micromeres at the animal pole divide and migrate vegetally as a sheet, enveloping the 4^th ^quartet micromeres and macromeres (Fig. 1H, I, J). It appears as though the micromeres 4a-c do not divide, but coalesce into the yolk filled interior of the embryo. The four small fourth quartet macromeres are visible and remain undivided until the ectoderm is established as a layer and just before it begins to invaginate at the lower pole to form the pharynx. By 95 hpo (48% d.t.) epiboly is almost complete and the animal-vegetal axis turns into the antero-posterior axis.

**Figure 2:**
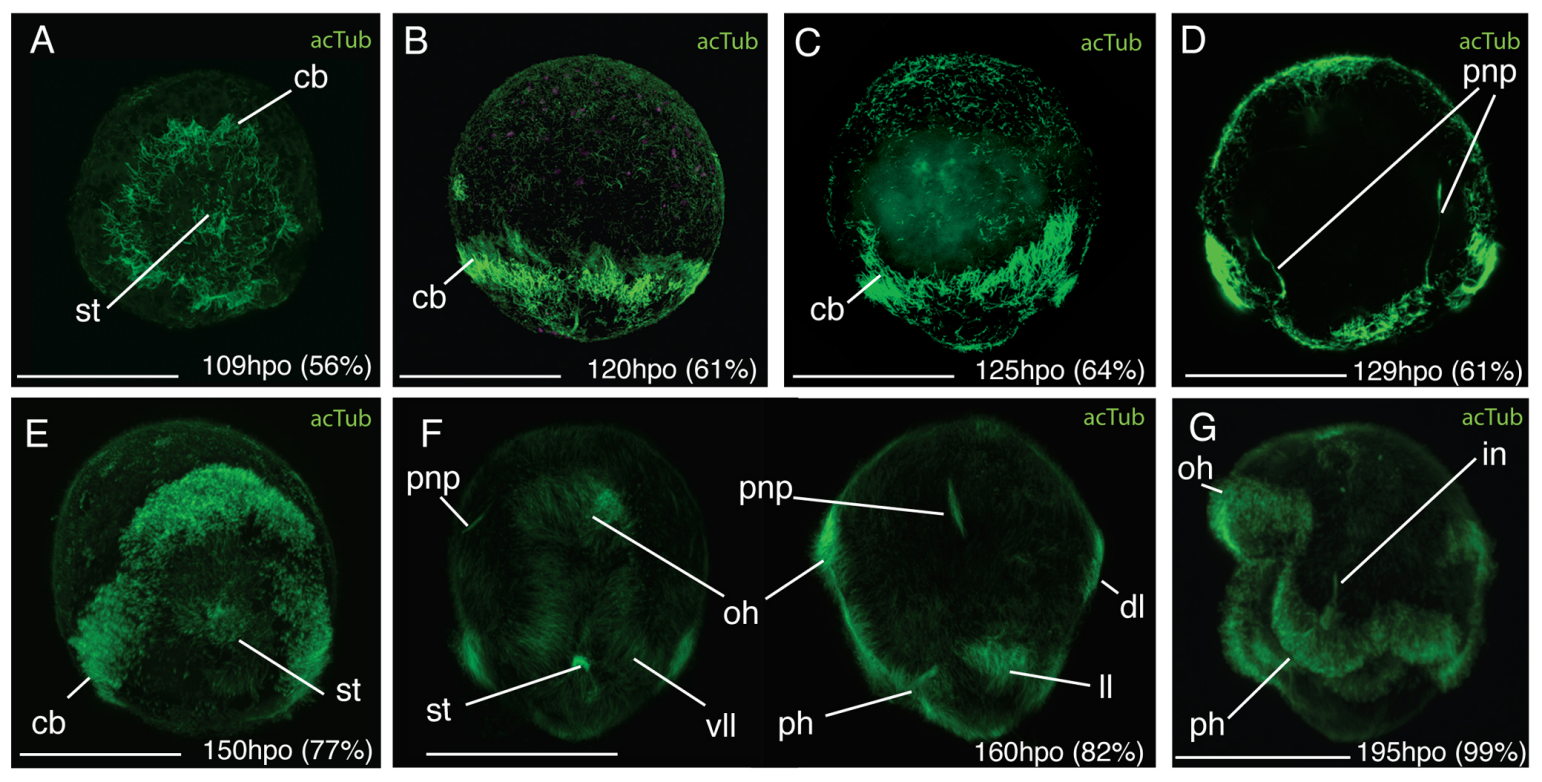
**Ciliary band migration in *Maritigrella crozieri *embryos**. CLSM micrographs of post-gastrulation acetylated tubulin stained wholemounts. **(A) **Posterior view showing ciliary ring around stomodeum (*st*) at posterior pole and the early ciliary band (*cb*). **(B-C) **Lateral view - anterior up - (B) ciliated epidermis and sub-equatorial ciliary band, (C) four branches of ciliary band migrate anteriorly, **(D) **Ventral view at focal plane of developing protonephridial canal cells (*pnp*). **(E) **Ventral view - stomodeum migrates ventrally, **(F) **Ventral and left lateral view - ciliary band demarcates position where the eight larval lobes will form (oral hood, *oh*, ventro-lateral lobe, *vll*, lateral lobe, *ll*, dorsal lobe, *dl*), cilia in the protonephridia lumen (*pnp*) and pharynx (*ph*) are visible. **(G) **Left lateral view-pre-hatchling showing continual band of longer cilia round the lobes and fused intestine (*in*) and pharynx. Scale bars 100 μm

Following gastrulation, development may be considered in three main stages; i) development of the epidermis and organ primordia, ii) organ differentiation, iii) development of larval lobes. Development of the epidermis and organ primordia takes place between 69 and 125 hpo (35% and 63% d.t.). During this time the embryo is round in shape and a small outpocketing develops at the posterior end, in the centre of which lies the stomodaeum. Smooth epithelial ectodermal cells are differentiated from more rounded deep cells. At 69 hpo (35% d.t.), epidermal cilia stain positive for acetylated tubulin, and rotation of the embryos starts at 75 hpo (38% d.t.). At 109 hpo (56% d.t.) a pre-oral ciliary band forms a circle sub-equatorially around the posterior end of the embryo and a ring of longer cilia forms around the developing stomodaeum (Fig. 2A). Sixteen hours later, the ciliary band has four offshoots which branch upwards towards the anterior of the embryo (Fig. 2C). The epidermal eyespot forms at 115 hpo (58% d.t.), in an anterio-dorsal position.

From 126 hpo (64% d.t.) to 169 hpo (86% d.t.) organ differentiation takes place and the embryo becomes obovate by elongating slightly along the antero-posterior axis. During this period two acetylated tubulin (acTub) expressing structures are visible, these are the two developing protonephridial canal cells (129 hpo, Fig. 2D). They extend from either side of the stomodeum, across the epithelium and basement membrane and proceed anteriorly. The two putative terminal cells of the protonephridia are visible in acTub stained wholemounts later (160 hpo) as two bilaterally symmetric, unbranched, elongated and heavy ciliated vesicles, located in the upper third of the embryo either side of the oral hood (Fig. 2F). The left cerebral eye develops first (139 hpo [70% d.t.]), followed shortly by the right cerebral eye, they are 20 μm from each other and are positioned more ventrally relative to the epidermal eye. The stomodaeum migrates from a posterior position to a mid-ventral position at 150 hpo (Fig. 2E), the pharynx invaginates later at 160 hpo (Fig. 2F), while the ciliary band migrates equatorially and demarcates the position where the eight larval lobes will develop (Fig. 2E). The F-actin containing microtubular sheaths of rhabdite cells are visible at the anterior and posterior tip of the embryo and become more numerous.

**Figure 3:**
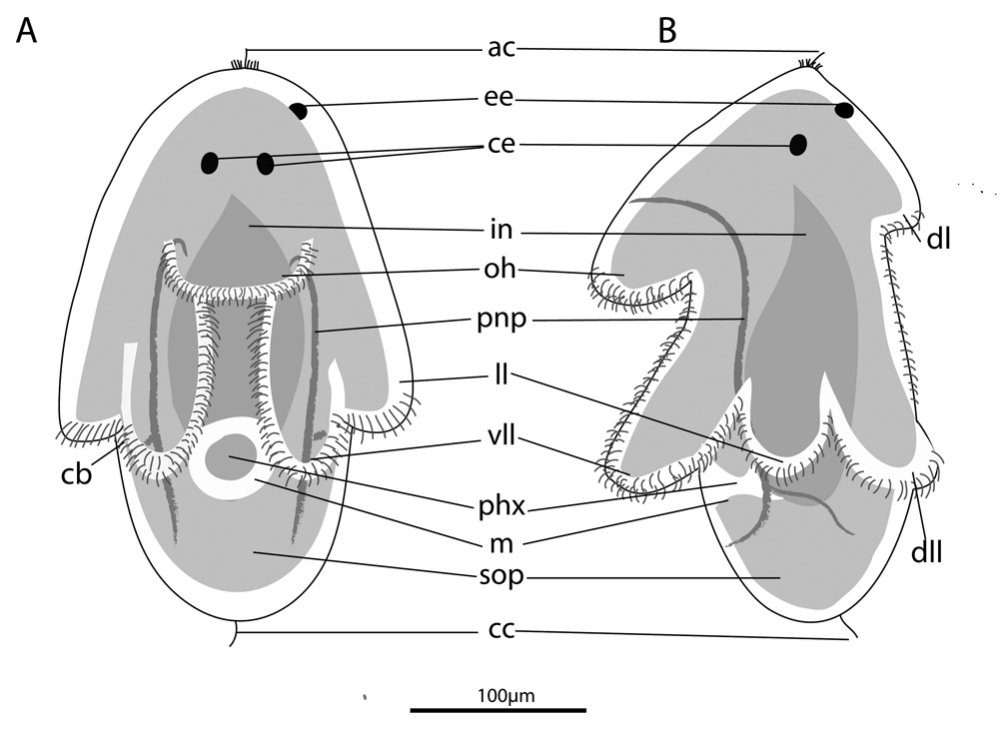
**Gross anatomy of *Maritigrella crozieri *two-day post-hatching pelagic stage**. A) ventral view, B) left lateral view. (*ac*) apical cilium, (*ee*) epidermal eye, (*ce*) cerebral eye, (*in*) intestine, (*oh*) oral hood, (*pnp*) protonephridia, (*ll*) lateral lobe, (*vll*) ventro-lateral lobe, (*ph*) pharynx, (*m*) mouth, (*sop*) sub-oral plate, (*cci*) caudal cilium.

From 170 hpo (87% d.t.) to 196 hpo (100% d.t.) the embryo's shape changes from obovate to a complex morphology with eight protruding lobes. Lobe formation starts with the protrusion of the oral hood (170 hpo [87% d.t.]) followed by the dorsal lobe. The paired lobes follow sequentially with the ventro-lateral lobes (175 hpo, 89% d.t.), followed by the lateral lobes and shortly after, the dorso-lateral lobes (which elongate further after hatching). The lateral lobes protrude higher up the embryo than the ventolateral and dorsolateral lobes. The pharynx and gut lumen meet at 175 hpo (89% d.t.). The gut is blind, ciliated throughout, un-branched (Fig. 2G) and reaches almost to the latitude of the brain in the developed larva. Hatching occurs on day 8 after 196 hours of embryonic development. The larva is positively phototactic and swims in a right-handed helix - led by epidermal eye (ie anterio-dorsal side leading). It is planktotrophic [26] and was observed to feed on cultures of unicellular algae introduced into the seawater (pers obs). Rhabdites, and pigmentation do not extend onto the lobes. The protonephridia extend anteriorly from below the mouth through the basement membrane, branching mid-larvae at the level of the lateral lobes, and re-enter the epidermis ventro-anteriorly just above the oral hood at the same latitude as the brain. They run interior to, but in close proximity with, the lateral nerve connectives. The average size of a hatchling is 250 (± 12, *n *= 10) μm in length and 200 (± 8, *n *= 10) μm wide. The gross anatomy is illustrated at two-day post hatching (Fig. 3).

### Larval muscle anatomy

**Figure 4:**
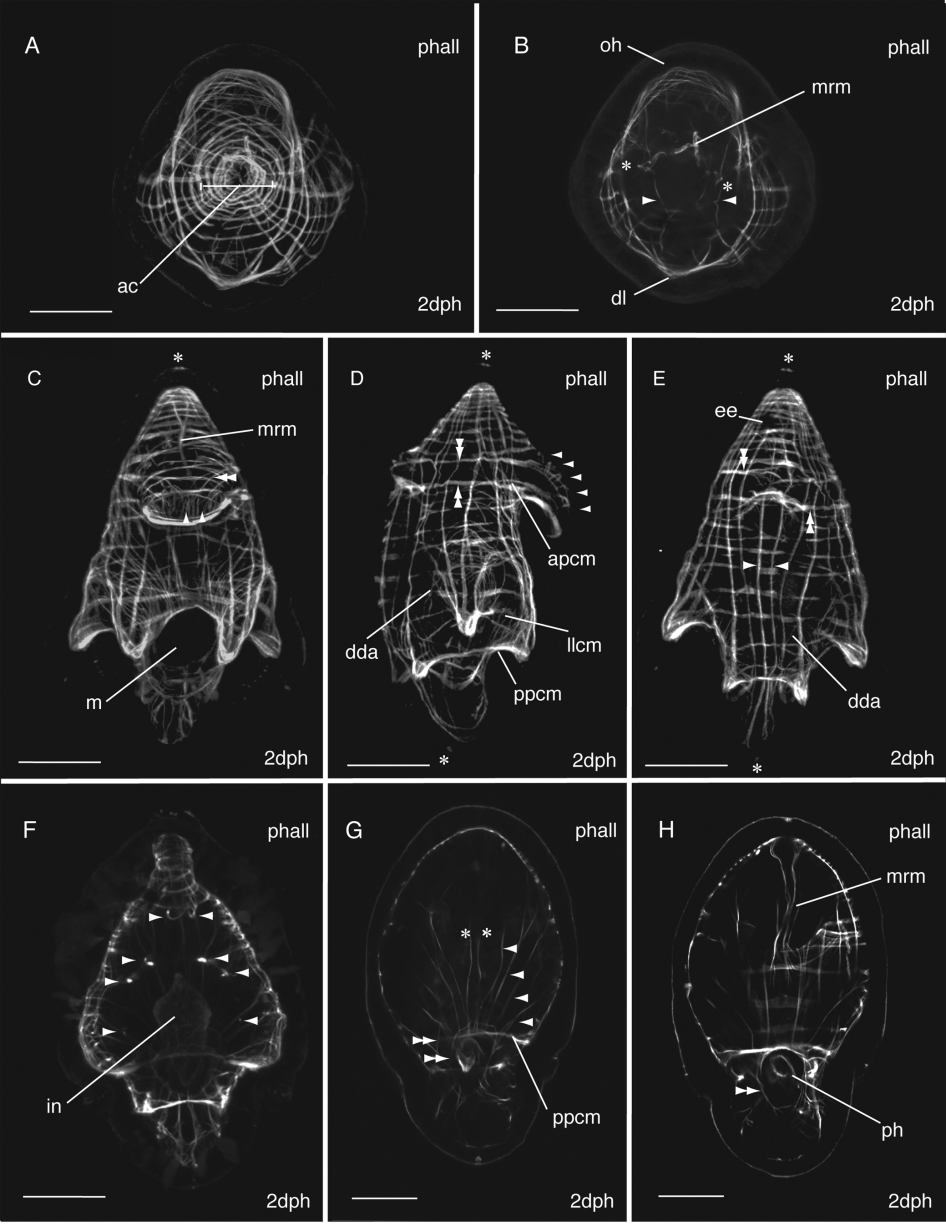
**Confocal projections of the phalloidin-labelled musculature of *Maritigrella crozieri *two-day post-hatching pelagic stage**. Apical views (ventral up) of **A) **Apical complex (*ac*) of circular and coiled muscles above the orthogonal grid of body wall muscles, and **B) **optical sections below the apical complex showing mouth retractor muscle (*mrm*) and a pair of anterior-posterior parenchymal muscles (asterisk) extending from the apical complex to the mouth (*m *in C). A pair of dorso-ventral parenchymal muscles (arrow heads in B) extends from the dorsal lobe (*dl*) to the oral hood (*oh*). **C) **Ventral, **D) **lateral and **E) **dorsal view of orthogonal grid of body wall muscles, mouth retractor muscle (*mrm*) and oral hood muscles; longitudinal (arrowheads in **C)**, oblique (double arrowhead in **C**) and circular (arrowheads in **D)**. Prominent circular muscles include the anterior primary circular muscle (*apcm*), the posterior primary circular muscle (*ppcm*), and the lateral lobe circular muscle (*llcm*). Two thick muscle bands extend between the oral hood and dorsal lobe (double arrowheads in D & E), the endings branch obliquely across the lobes (double arrowhead in C) and interdigitate with endings from the opposing fiber. Primary longitudinal muscles (arrow heads in E), epidermal eye (*ee*). Roots of the apical and caudal cilia are visible in the epidermis (asterisks in C, D & E). **(F) **Dorsal view, optical sections inside of body wall musculature at focal plane of dorsoventral parenchymal muscles (arrowheads), three pairs in bilateral organisation lateral to the intestine (*in*). **(G) **Ventral view, single optical section showing many parenchymal muscles radiating from the posterior primary circular muscle (*ppcm*) laterally (arrow heads) and anteriorly (asterisk), concentric rings of mouth muscles (double arrow heads). **(H) **More ventral still, an optical section behind the ventral lobes shows the mouth retractor muscle (*mrm*) extending from the apical complex and a ring of muscle (double arrowheads) around the pharynx (*ph*). Scale bars 50 μm.

The body wall musculature at two-days post-hatching (n = 30)(Fig. 4) is composed of an apical complex on top of circular and longitudinal fibers organized in a lattice-like arrangement. Parenchymal muscles include dorso-ventral muscles and those associated with the mouth and pharynx.

The apical complex (Fig 4A), at the anterior pole of the larva, was made up of 5 spirally arranged coils or closed circular muscles. The apical coil had a diameter of 15 μm. These muscles serve as attachment sites for most of the longitudinal muscles. Approximately 6 longitudinal muscles met in the centre of the complex at the anterior pole. Below, an orthogonal grid of 12-22 outer circular and 12-16 inner longitudinal muscles constitutes the framework of the body wall musculature. Of these, two circular and two longitudinal muscles are considered primary muscle fibres, as they were the earliest distinguishable landmarks during myogenesis (see below). These muscles were more deeply set than the rest of the body wall muscle grid. The primary circular muscles (Fig. 4D) consist of an anterior primary circular muscle and the posterior primary circular muscle. The former follows the lower contour of the oral and dorsal lobes. The posterior primary circular muscle forms the lower muscle band of the ventro-lateral and dorso-lateral lobes, but is not connected to the lateral lobe, which protrudes higher up the larvae. However, it runs close to the lateral lobe circular muscle ventrally above the mouth. On the dorsal side two primary longitudinal muscles (Fig. 4E) run parallel to each other down from the apical complex to the caudal pole, under the sub-oral plate to join the mouth muscles on the ventral surface.

Bilateral symmetry of the longitudinal muscles is apparent from the apical view (Fig. 4A). 2-3 major longitudinal muscle fibres extend posteriorly from the apical complex into each lateral lobe. At the end of the lobes these muscles digitate into fine muscle fibres. Of these longitudinal muscles, the two that form the lateral ridge of the lateral lobes are particularly thick and prominent. Four longitudinal muscles on the dorsal side, including the two primary longitudinal muscles, pass under the sub-oral plate and are joined by many fine muscles leading to the mouth. The lateral lobe circular muscle (Fig. 4D) runs around the larvae at the level of the two lateral lobes and over the anterior opening of the mouth. Many of the prominent circular and longitudinal muscles appear to be double stranded. Many fine circular muscles converge at the mouth. A first diagonal body wall muscle (Fig. 4D, E) runs from a posterior medial position on the dorsal side around to the right lateral side, arcing up behind the lateral lobe and curves down towards the ventro-lateral lobe. This is named the dorsal diagonal arc in *Hoploplana inquilina *[19], and in *M. crozieri *it becomes more pronounced, extending from the left caudal side to the right side of the larvae as it develops. By 10 days post-hatching other diagonal fibres are visible including two short diagonal muscles that cross each other above the dorsal lobe (see additional file 1 figure S1a).

**Figure 5:**
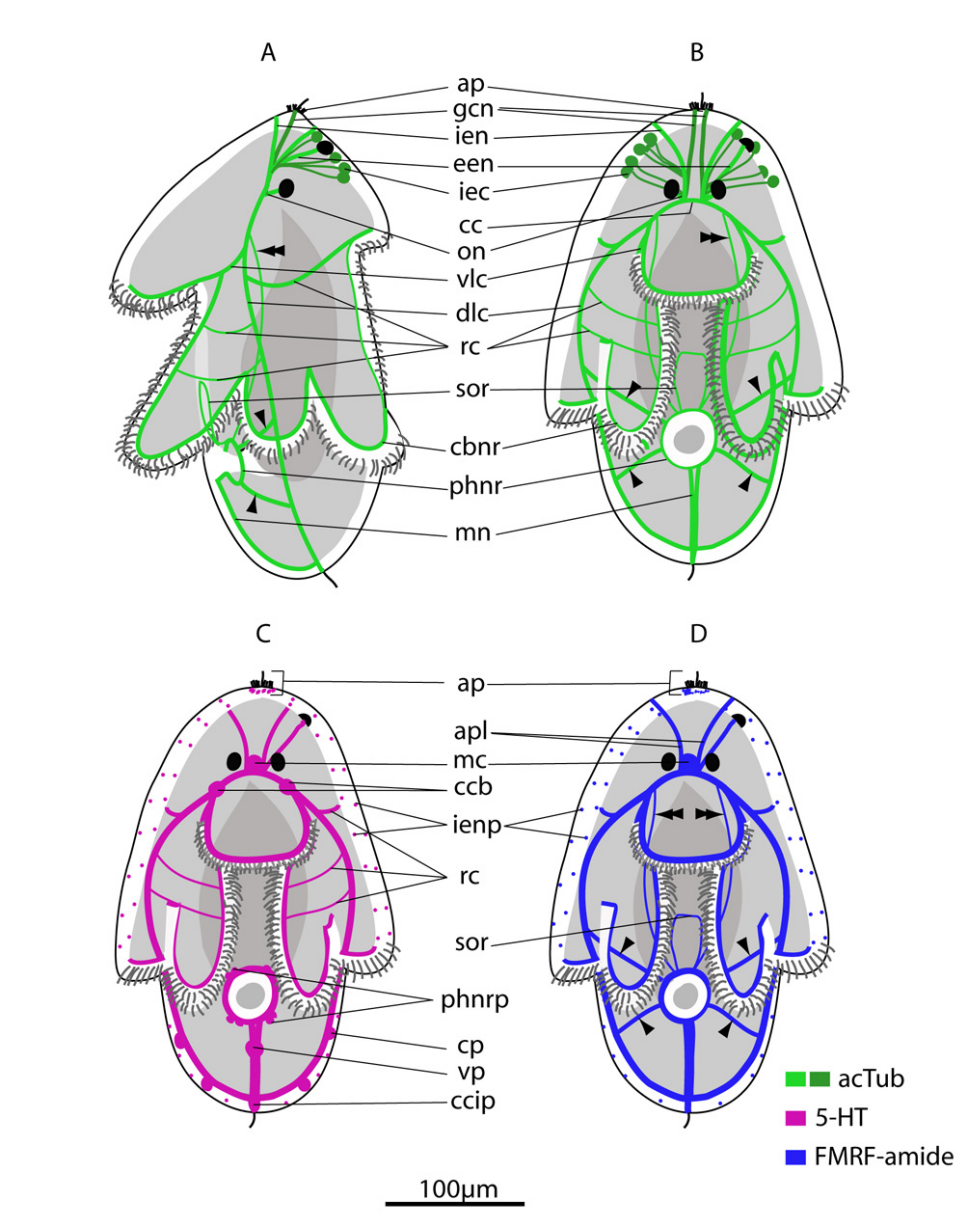
**The neural groundplan of the *Maritigrella crozieri *two-day post-hatching pelagic stage**. **(A & B) **based on acetylated tubulin wholemounts, left lateral and ventral view respectively (bright green = neurons that show serotonin and/or FMRFamide immunoreactivity, dark green = structures not immunoreactive to serotonin and FMRF-amide antibodies). (**C) **Serotonin expression - ventral view. (**D) **FMRF-amide expression - ventral view. (*aa*) apical plate, (*apl*) apical plexus, (*cbnr*) ciliary band ring, (*cc*) cerebral commissure, (*ccb*) commissural cell bodies (*ccip*) caudal cilium perikarya, (*cp*) caudal perikarya, (*dlc*) dorso-lateral connective, (*een*) epidermal eye nerve, (*gcn*) gland cell necks, (*iec*) intra-epidermal cells, (*ien*) intra-epidermal nerves, (*ienp*) intra-epidermal nerve plexi, (*mc*) medial cluster, (*mn*) medial nerve, (*on*) oellar nerve, (*phnr*) pharyngeal nerve ring, (*rc*) ring connectives, (*sor*) supra-oral ring, (*vlc*) ventro-lateral connective, (*vp*) ventral perikarya.

**Figure 6:**
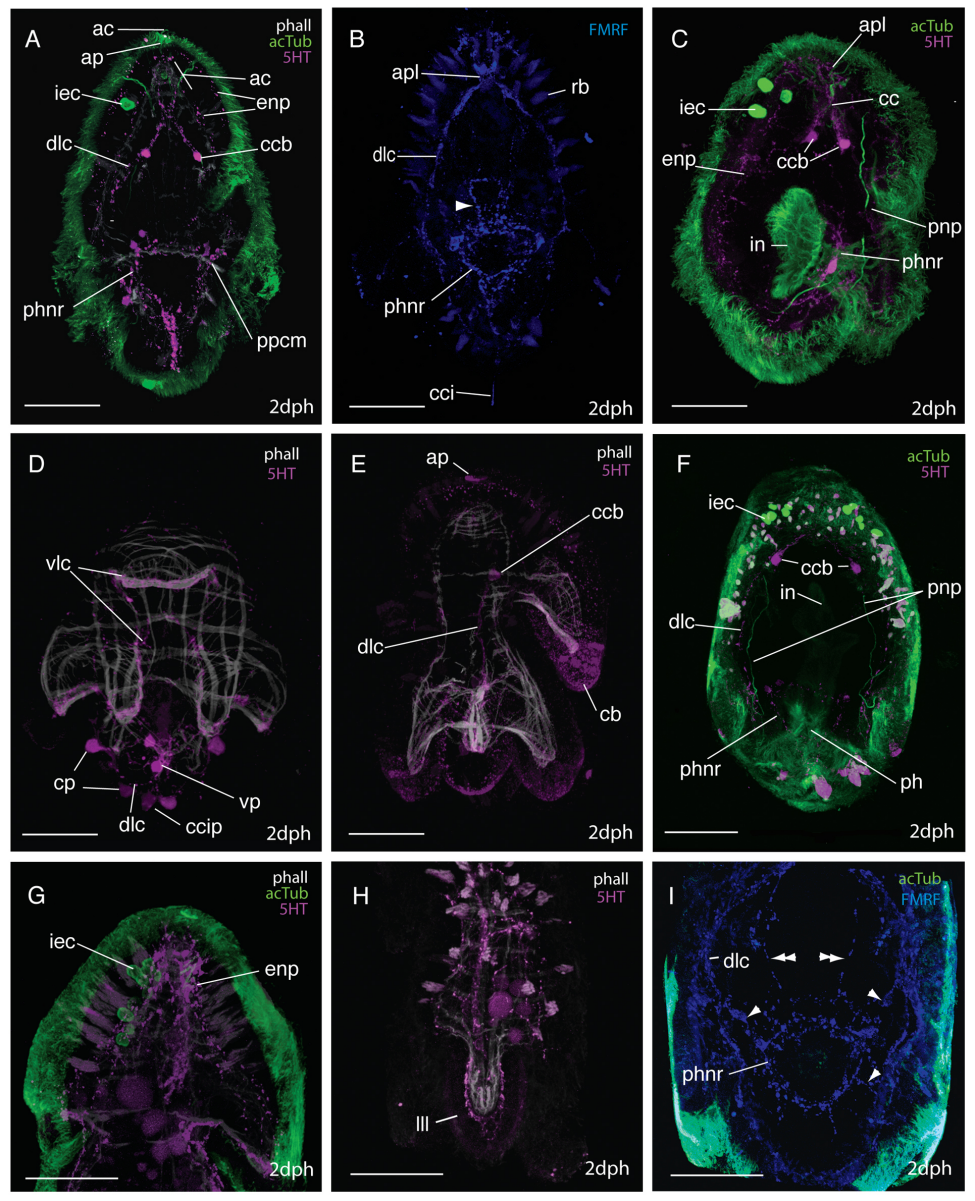
**CLSM micrographs of the nervous system in two-day post-hatching *Maritigrella crozieri***. (phalloidin-white, acTub-green, 5HT-magenta, FMRFamide-blue [except in C where 5HT and FMRFamide-magenta]). **(A) **Mid optical sections of triple labeled larva (ventral view), showing neural components; commissural cell bodies [*ccb*], dorso-lateral connective [*dlc*], pharyngeal nerve ring [*phnr*], intra-epidermal cells [*iec*], epidermal nerve plexi [*enp*], apical plate [*ap*] and apical cilium [*ac*], in relation to the apical complex muscles (*ac*) and posterior primary circular muscle (*ppcm*). **(B) **FMRFamide expression in the apical plexus (*apl*), dorso-lateral coonective, pharyngeal nerve ring and supra-oral nerve ring (arrowhead) Ventral view. Rhabdites (*rb*). **(C) **Dorso-lateral view showing the neural activity in the apical plexus, the cerebral commissure (*cc*) and commissural cell bodies, and the spatial relationships between the intra-epidermal cells, epidermal nerve plexi, pharyngeal nerve ring, protonephridia (*pnp*), and intestine (*in*). **(D & E) **Ventral and lateral views of the nerve and muscle orthogons showing the ventro-lateral (*vlc*) and dorso-lateral connectives, the innervation of the sub-oral plate (caudal perikarya (*cp*) and caudal cilia perikaryon (*ccip*) on the dorso-lateral connective and ventral perikarya (*vp*) on the medial nerve), and the intra-epidermal 5HT expression at the apical plate and the ciliary band (*cb*). **(F) **Dorsal view showing the spatial relations of the protonephridia (*pnp*) and dorso-lateral connectives, the pharyngeal nerve ring and the pharynx (*ph*) and intestine, and the intra-epidermal cells and the neuropile and commissural cell bodies. **(G) **Right lateral view of head, 5HT expression in epidermal nerve plexus external to the apical complex of body wall muscles and acTub IR in intra-epidermal cells. **(H) **Neuromusculature of left lateral lobe (lll) showing nerves external to body wall muscles. **(I) **Ventral view of FMRFamide expression in the pharyngeal nervous system shows connections between the pharyngeal nerve ring and the dorso-lateral connectives (arrowheads) and cerebral commisure (double arrowheads). Scale bars 50 μm.

**Figure 7:**
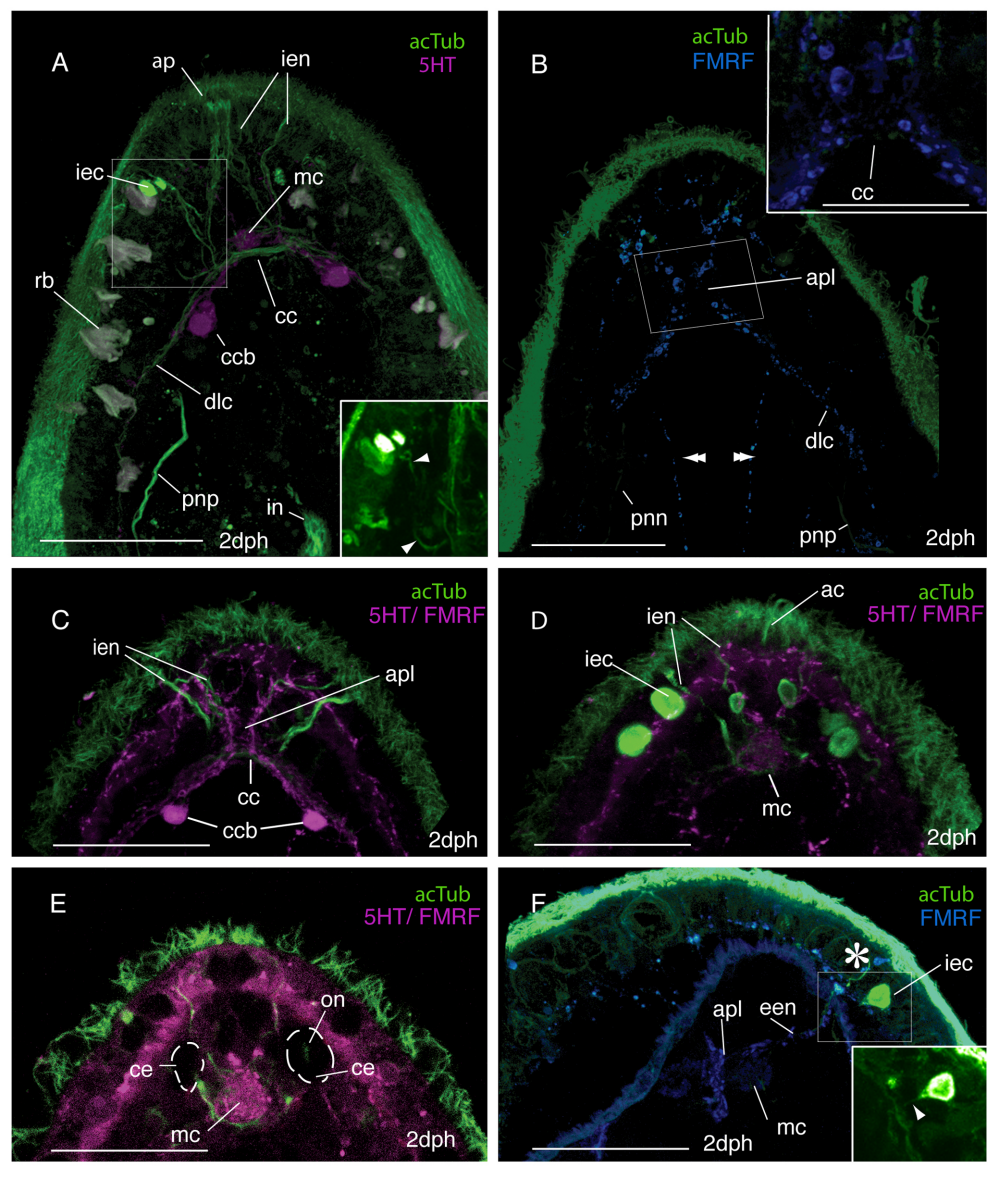
**The larval brain of *Maritigrella crozieri *- CSLM micrographs of neural components**. (A-E) Ventral views of the anterior end of the larvae. **(A) **AcTub (green) and 5HT (magenta) expression showing the posterio-lateral extension of the dorso-lateral nerve cords (*dlc*) and anterior and anterior-lateral extension of intra-epithelial neurites (*ien*) to the apical plate (*ap*) and surface from the cerebral commissures (*cc*). 5HT expression is pronounced in the commissural cell bodies (*ccb*) and median cluster (*mc*). The protonephridia (*pnp*), intra-epidermal cells (*iec*) and cilia in the intestine (*in*) show acTub IR and rhabdites (*rb*) are visible in the epidermis. Inset shows neural connection (arrow heads) between the intra-epidermal cells and the cerebral commisure. **(B) **FMRFamide (blue) and acTub (green) expression, note lack of FMRFamide expression in commissural cell bodies but immunoreactivity in the apical plexus (*apl*) above the cerebral commissure and in two nerves connecting the brain to the pharynx (double arrowheads). Inset shows FMRFamide and acTub expression in cerebral commissure. (**C, D, E**) Dual labeling of acTub (green) and FMRFamide and 5HT simultaneously (magenta) showing; **(C) **ventral section of brain with commissural cell bodies, cerebral commissure, apical plexus and intraepithelial neurites, **(D) **dorsal section of brain consisting of median cluster and intraepithelial neurites in close proximity to intra-epidermal cells (*iec*) and apical cilia (*ac*). **(E) **Optical section at the focus plane of the cerebral eyes (*ce*) either side of median cluster, acTub expressing optic nerves (*on*) visible. **(F) **Left lateral view of anterior end of larva showing FMRFamide (blue) expression in the median cluster dorsal to the apical plexus. FMRFamide and acTub IR is seen in epidermal eye nerve (*een*) extending dorsally to the epidermal eye (asterisk) Inset shows neuron (arrowhead) from intra-epidermal cell passing through the basement membrane. Scale bars 50 μm, inset in B 25 μm.

**Figure 8:**
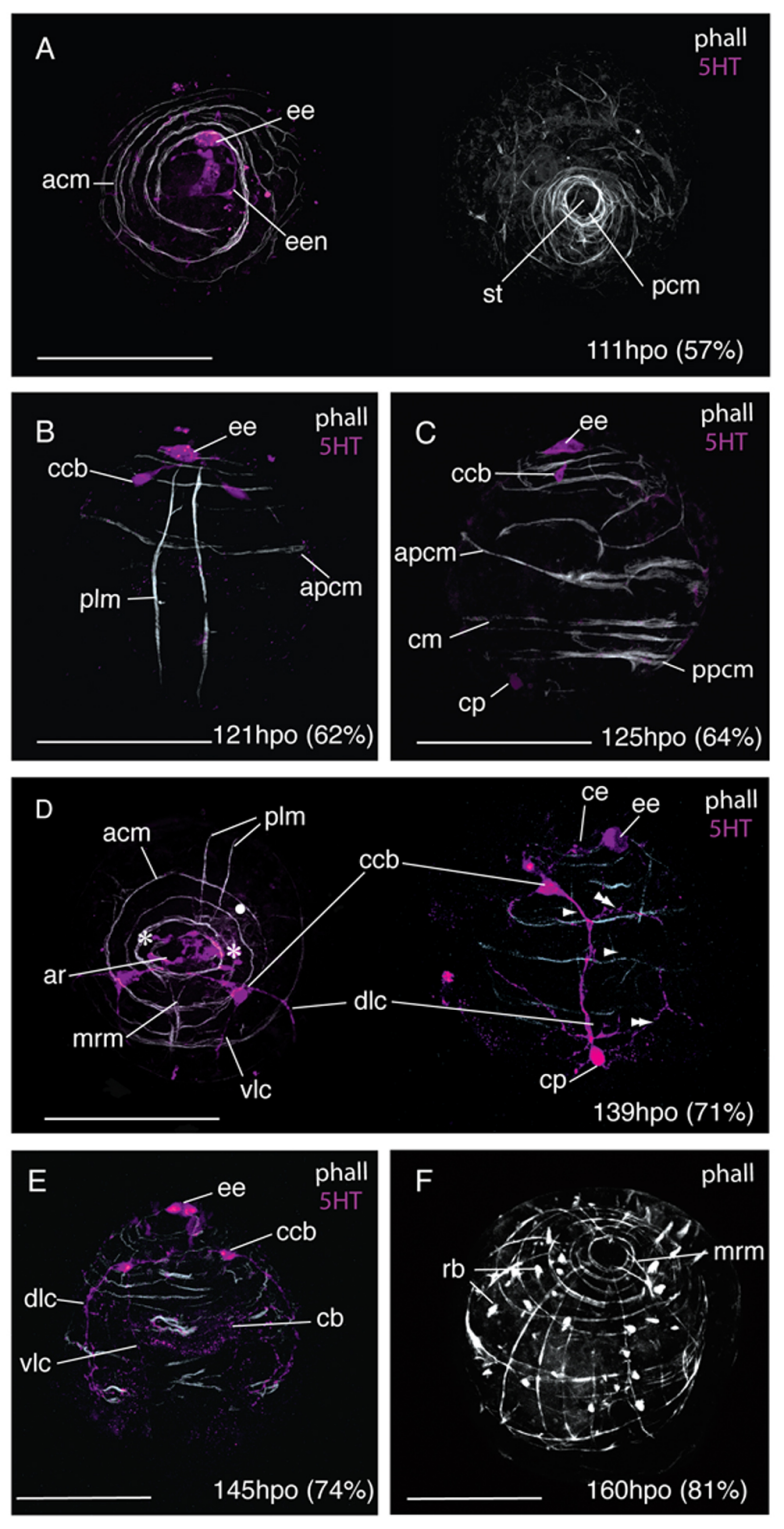
**Early development of the nervous and muscle systems in *Maritigrella crozieri***. CLSM micrographs of fluorescently labeled wholemounts from 55-70% development time (phalloidin - white, 5HT - magenta). **(A) **Left - anterior view, right - posterior view. Early development of apical circular myofibrils (*acm*) and posterior circular muscles (*pcm*) around the stomodeum (*st*). 5HT immunoreactivity in the developing epidermal eye (*ee*) and it's associated nerve (*een*). **(B) **Dorsal view showing the primary longitudinal muscles (*plm*) and their position relative to the epidermal eye, apical circular muscles and anterior primary circular muscle (*apcm*). Rudiments of the commissural cell bodies (*ccb*) are developing. **(C) **Left lateral view showing further development of circular muscles (*cm*) and 5HT expression in caudal perikarya (*cp*). **(D) **Anterior and left lateral view show neuropil condensation. An apical ring (*ar*) of 5HT expressing cells in the epidermis sits between the cerebral eyes (asterisks) and above the apical circular muscles, the commissural cell bodies and nerves are below the body wall muscles. The dorso-lateral (*dlc*) and ventro-lateral (*vlc*) connectives migrate posteriorly and the ring commisures extend dorsally (double arrowheads). Several longitudinal muscles (arrowheads) form and early mouth retractor muscles (*mrm*) grow from the apical complex posterior-ventrally to the mouth. **(E) **Ventral view shows predominance of circular muscles and the first signs of 5HT expression under the ciliary band (*cb*). **(F) **Anterio-lateral view shows the establishment of the orthogonal body wall musculature with the development of up to 12 longitudinal muscles. The mouth retractor muscles (*mrm*) are visible on the ventral side and increasing numbers of rhabdites (*rb*) develop in the epidermis Scale bars 100 μm.

The musculature of the concave oral hood consists of 5-7 circular muscles (Fig. 4D) and 3-8 oblique muscle fibres (Fig. 4C) radiating from paired thick bands of muscle on either side of the hood (Fig. 4D). These paired muscles bands run from the upper surface of the dorsal lobe to the oral hood. A second pair extends (Fig. 4D) along the lower margin of the dorsal and oral lobes, and they converge ventrally with the anterior primary circular muscle and a lower circular muscle to form the thick, prominent muscle at the front of the lobe. There are 9 fine longitudinal muscles (Fig. 4C) in the oral hood that are positioned inside of the circular and oblique muscles. These muscles connect to the upper margin of the mouth muscles. The dorsal lobe is made up of four circular muscles (including the anterior primary circular muscle) and the two pairs of thick muscle bands that wrap around the larva from the oral hood to the dorsal lobe. The endings branch and interdigitate on the dorsal lobe protrusion (Fig 4E). The three pairs of lateral lobes that protrude from the body wall each have 2-3 longitudinal muscles and several circular muscles. Numerous fine circular and diagonal muscles are found along the length of the lobes and at the tips.

There are five pairs of bilaterally symmetrical parenchymal dorsoventral muscles; three run lateral to the intestine, between the mouth and anterior primary circular muscle (Fig. 4F). The fourth pair lies inside of the anterior primary circular muscle and joins the dorsal lobe to the oral hood (Fig. 4F). The fifth pair is more anterior and is at the same latitude as the 8^th ^circular muscle from the apex (Fig. 4B). Several more dorsoventral parenchymal muscles are found in the sub-oral plate. Six - twelve diagonal parenchymal muscle fibres run anteriorly from the posterior primary circular muscle just above the mouth margin, two connect below the apical complex, several run to the frontal muscle of the oral hood and the others connect to circular muscles laterally below the latitude of the cerebral eyes (Fig. 4G). These diagonal muscles probably act as mouth dilator muscles. The mouth retractor muscle (Fig 4B, C, H) bifurcates at the anterior pole of the larva, each branch attaches to either side of the apical complex. Just below the cerebral eyes it forks into several branches, which divide into many fingers. Some of these fingers reach down to the mouth circular muscles, while others attach to the lower muscles of the oral hood. Three ring-shaped fibres form a sphincter at the mouth (Fig. 4G and 4H). F-actin in the cilial band was stained with phalloidin and two rows of cilia were observed on the oral and ventro-lateral lobes, while only one is observed on the lateral, dorso-lateral and dorsal lobes (additional file 1 figure S1 b).

### Larval neuroanatomy

The ground pattern of nerves at two-days post-hatching (n = 50) is described based on acetylated tubulin (acTub) stained wholemounts (Fig. 5A &5B). Acetylated tubulin marks stabilized microtubules that form in, for example, axon fibers and gland cell necks. In order to begin to distinguish neural from non-neural elements, the distribution of the neurotransmittors serotonin (5HT) and FMRF-amide was mapped onto the ground pattern. Neurons that showed immunoreactivity to the neurotransmittors were located in the apical plate, the neuropile, paired dorso-lateral and ventro-lateral nerve cords, a pharyngeal nerve ring, a medial nerve, a ciliary band nerve ring, and two intra-epidermal nerve plexi (Fig. 5C &5D). Many acTub labeled structures did not show 5HT or FMRFamide immunoreactivity, indicating that other neurotransmittors might be active or that they are non-neural structures (see below, Fig. 5).

The apical plate is located above the apical spiral complex of the body wall muscles (Fig. 6A) and consists of: 1) a single apical cilium (Fig. 6A, 7A), 2) a ring of longer cilia surrounding the apical cilium (Fig. 6A) and 3) a ring of 5HT (Fig. 6E) and FMRFamide expression beneath the ring of long cilia. Whether the ring of long cilia are from cells that are 5HT and FMRFamide positive or whether these epidermal ciliated cells are non-neuronal but are in contact with dendrites of 5HT and FMRFamide positive cells is not discernable from this data and requires further investigation. Indeed how the apical plate is connected to the neuropile is not evident from these data. The acTub structures leading from the apical plate to the cerebral commissure are not 5HT or FMRFamide-positive and it is possible that they could be the microtubules in the gland cell necks (Fig 7A).

Intraepithelial neurons surrounding the apical plate extend posteriorly between the body wall muscles to a cluster of axons - the apical plexus, which is situated in the midline, anterior to, but in contact with, the cerebral commissure and forms part of the neuropile (see below)(Fig. 7A, D, E, F). The brain of the larvae consists of a bilaterally symmetric central neuropile made up of two distinct regions; the apical plexus and the cerebral commissure. The apical plexus consists of a rectangular shaped network of FMRF-amide expressing cell processes (Fig. 7B) and the spherical median cluster expressing 5HT and FMRFamide - this may be a synaptic glomeruli where the sensory afferents innervating the apical plate, intra-epidermal cells and other intra-epidermal neurites converge (Fig. 7C). Nerves expressing both neurotransmittors proceed from the apical plexus to the epidermal eye (Fig. 7F) (5HT data not shown). The cerebral commissure consists of an orderly array of neurons, with subsets showing immunoreactivity to FMRF-amide and 5HT (Fig. 7A, B). Two very distinct 5HT-expressing cell bodies sit either side of the cerebral commisure (Fig. 6A, C, E, F and 7A, C). Neurons from the commissure can be traced into all four main body nerve cords; the paired ventro-lateral and dorso-lateral nerves (Fig. 6A, B, D, E F &7A, B, C). The two cerebral eyes sit either side of the apical plexus and acTub visible fibres lead from the cerebral commissure to the eyes (possible ocellar nerves) (Fig. 7E).

The dorso-lateral and ventro-lateral nerves (5HT, FMRFamide IR) diverge at the junction of the prominent 5HT-expressing commissural cell bodies. The ventro-lateral nerves extend ventrally, and branch at the level of the anterior primary circular muscle. One branch aligns with this muscle around the oral hood, while the second branch follows the inner margin of the ventro-lateral lobes (Fig. 6D). The dorso-lateral nerve cord extends posterio-laterally (Fig. 6A, E, 7A, B) until it reaches the body wall muscles where it aligns with the prominent lateral longitudinal muscle. Projections branch off the dorso-lateral nerve cord dorsally with many following the contours of circular muscle bands. Below the lateral lobe the main branch of the dorso-lateral nerve sits external to the bodywall musculature and meets its pair at the caudal cilium (Fig. 6B). The caudal cilium is slightly dorsal to the posterior pole, and is located beneath the two primary longitudinal muscles (Fig. 6D). One neuronal cell body (5HT and FMRFamide IR) innervates the caudal cilium, and two more perikarya are located on both sides of the caudal tuft on the dorso-lateral nerve cord (Fig. 6D).

From the caudal cilium, two medial FMRFamide and 5HT expressing nerves extend to a ventral perikarya before diverging and connecting to the pharyngeal nerve ring (Fig. 6B, D). Several 5HT and FMRFamide expressing nerves (Fig. 6B, C, F, I) form a net around the concentric rings of the pharyngeal musculature (Fig. 6A, D). Numerous (2-5) bilaterally symmetric perikarya are visible on this nerve ring (Fig. 6A, C, F). Many nerves (2-4) radiate laterally from each side of the pharyngeal ring to join the dorso-lateral nerves and two nerves join the cerebral commissure at the commissural cell bodies (FMRFamide IR only) (Fig. 4I). No nerves or plexi were observed around the intestine. Anterior to the pharyngeal nerve ring sits a ring of FMRF-amide expressing cells (Fig. 6B). This is located intra-epidermally between the ventro-lateral lobes.

A grid of nerves that express FMRFamide and 5HT innervate the musculature of the larval lobes. The nerves are external to the musculature and circular (ring or transverse) nerves form commissures connecting the four main nerve cords (Fig. 6D and 6H). 5HT and FMRFamide expression reveal two putative intra-epidermal nerve plexi; the first beneath the cilia and the second external to the body wall musculature (Fig. 6A, E, G and 7F), though where this second plexus sits in relation to the basement membrane needs verification at the ultrastructural level. 5HT and FMRFamide expression is particulary dense below the ciliary band with many transmittor release sites between the lobe muscles and ciliated cells of the ciliary band (Fig. 6E, additional file 1 figure S1c. FMRFamide data not shown).

A comparison of the aminergic (5HT) and peptidergic (FMRFamide) components of *M. crozieri *larval nervous system reveals there is significant overlap in immunoreactivity in the neuropil, dorso-lateral and ventro-lateral connectives, pharyngeal nerve ring and peripheral nerve plexus (especially under the ciliary band), though colocalisation was not tested. However only FMRFamide immunoreactivity was detected in the supra-oral ring and in portions of the pharyngeal nervous system, such as nerves leading to the dorso-lateral connectives and brain (Fig. 5D). Only 5HT immunoreactivity was detected in the two cell bodies at each side of the cerebral commissures (Fig. 5C).

Many acTub labeled structures did not show 5HT or FMRFamide immunoreactivity; the fibres leading to the cerebral eyes and structures leading from the neuropile to the apical plate (these have been discussed above). A prominent third feature identified by acTub staining was two clusters of 2 - 8 intra-epidermal cells at the anterior pole of the larvae (Fig. 6G). These clusters are located dorso-laterally to each cerebral eye, and a thin process extends from each cell through the basement membrane to the brain (Fig. 7A &7F). These cells are similar in their acTub expression to the 'apical gland cells' described in *Imogine mcgrathi *[21] and may correspond to the intraepithelial multiciliated cells described in *Pseudoceros canadensis *[24]. They are hypothesized to be ciliary photoreceptors (see discussion).

### The ontogeny of the nervous and muscle systems

#### 1) 0-125 hpo (0-63% d.t.)

**Figure 10:**
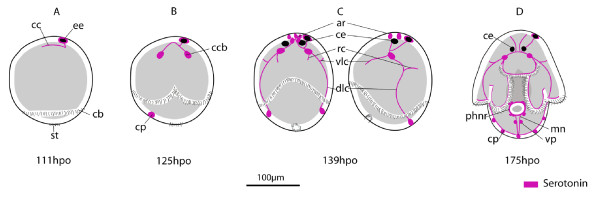
**Embryonic development of the serotonergic nervous system in *Maritigrella crozieri***. **(A) **111 hours post-oviposition (hpo) - ventral view, **(B) **125 hpo - ventral view, **(C) **139 hpo - i) ventral view, ii) left lateral view, **(D) **175 hpo - ventral view. (*ar*) apical ring, (*cb*) ciliary band, (*cc*) cerebral commissure, (*ccb*) commissural cell bodies, (*ce*) cerebral eye, (*cp*) caudal perikaryon, (*dlc*) dorso-lateral connective, (*ee*) epidermal eye, (*mn*) medial nerve, (*phnr*) pharyngeal nerve ring, (*rc*) ring commissures, (*st*) stomodaeum, (*vlc*) ventro-lateral connective, (*vp*) ventral perikarya.

During the first 54% of development time, F-actin was only visible in zonulae adhaerentes of epidermal cells. Approximately 12 hours after gastrulation, between 107 -119 hpo (55- 60% d.t.), the first muscles developed; 2-3 spiral coiled muscles at the anterior pole, plus a ring of interweaved circular muscles at the posterior pole surrounding the stomodeum (Fig. 8A). The first immunoreactive elements of the nervous system were a 5HT expressing ectodermal cell at the site of the developing epidermal eye, and a single neuron that connected this cell to further 5HT immunoreactive cells in the apical epidermis (Fig. 8A &10A). The two primary longitudinal muscles formed subsequently (121 hpo, 62% d.t.), 40 μm apart from each other and running parallel from the posterior pole to the apical circular muscles. They ran close to the epidermal eye indicating a dorsal position (Fig. 8B). Oblique muscles branching off from the longitudinal muscles developed into circular muscles, including the anterior primary circular muscle (Fig. 8B). Further circular muscles developed at the posterior pole and equatorially (Fig. 8C). Myogenesis appears to proceed by branching or fission of the pioneer myofibrils of the apical complex and primary circular and longitudinal muscles, many of which had forked ends (Fig. 8C). The two 5HT-expressing cell bodies of the cerebral commissure appear at 62% d.t. in close proximity to the epidermal eye, and 5HT expression is detected in a caudal perikaryon (Fig. 8C).

#### 2) 126-169 hpo (64-86% d.t.)

The first cerebral commissure labeled with acTub was observed at 126 hpo (64% d.t.) and from this extended one intra-epithelial neuron to the apical plate (not shown). At this stage 5HT expression was also visible in 1-2 caudal perikarya in the epidermis (Fig. 8C, D). By 139 hpo (70% d.t.) the cerebral eyes in the epidermis showed 5HT immunoreactivity. A ring-shaped cluster of 5HT expressing cells (12-14) formed in the apical epidermis between the cerebral eyes above the apical spiral complex of muscles (Fig. 8E). This signaled the start of brain condensation as further development of the commissural cell bodies and axons ensued below the body wall muscles. Migrations of the dorso-lateral and ventro-lateral connectives from the commissural cell bodies commenced. The former was thicker and more prominent than the later, and they grew out from the brain before the main period of longitudinal muscle growth. Early traces of dorsal and ventral circular commissures were visible. Faint 5HT immunoreactivity under the cilial band was detected (Fig. 8D). By 74% development time a third caudal perikarya had formed along the dorso-lateral connective in the suboral plate and 5HT expression was strong in the epidermis under the developing ciliary band (Fig. 8E). The first acTub labeled intra-epidermal cell formed at 160 hpo (81% d.t.).

Further circular muscles developed during this period between the anterior primary circular muscle and the apical spiral complex. These single fibres appear to form the lower coils of the apical spiral complex closing to completely encircle the embryo. During this period the number of longitudinal myofibrils also grew within the body wall. The first parenchymal muscles, early mouth retractor muscles, were visible at 139 hpo (71% d.t.) and grew from the apical complex posterior-ventrally to the forming mouth (Fig. 8D). By 160 hpo (81% d.t.) up to 14 circular muscles and 12 longitudinal muscles formed around the embryo establishing an orthogonal muscle grid (Fig. 8F). A dense band of circular muscles developed at the latitude of the developing dorsal lobe and oral hood. One prominent equatorial circular muscle was evident.

#### 3) 170 hpo (87% d.t.) - hatching

**Figure 9:**
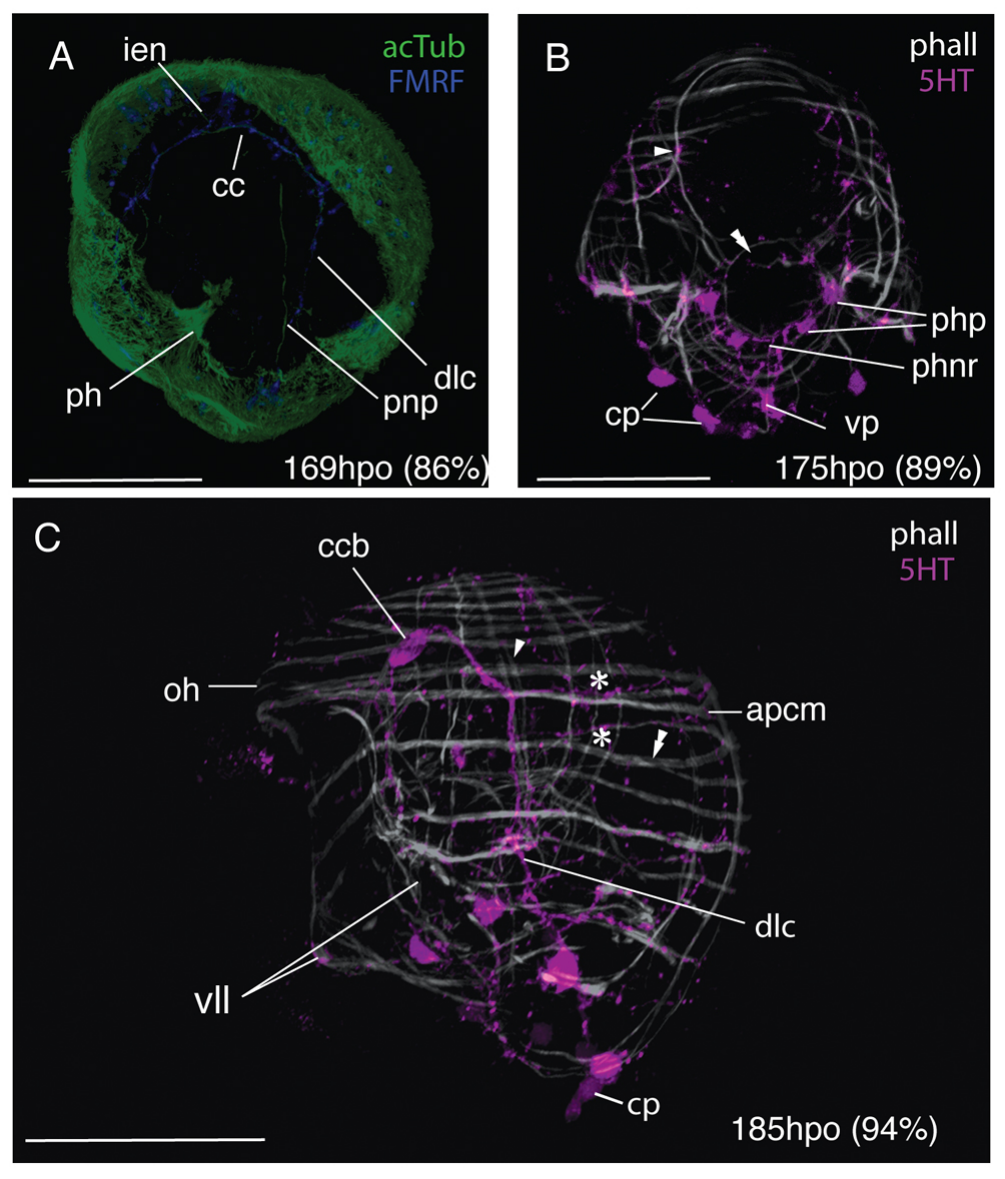
**Late embryonic development of the nervous and muscle systems in *Maritigrella crozieri***. CLSM micrographs of fluorescently labeled wholemounts from 71-100% development time (phalloidin - white, 5HT - magenta, acTub - green, FMRFamide - blue). **(A) **Ventro-left lateral view showing acTub and FMRFamide immunoreactivity in the cerebral commissure (*cc*), intraepithelial nerves (*ien*) and dorso-lateral connective (*dlc*). AcTub expression is also in the protonephridia (*pnp*) and in cilia in the pharynx (*ph*). **(B) **Ventral view showing circular muscle formation around the mouth (double arrowhead) and 5HT expression in the pharyngeal nerve ring (*phnr*) with its bilaterally symmetric perikarya (*php*). Two nerves extend posteriorly from the pharyngeal nerve ring and converge at a ventral perikarya (*vp*) before joining the dorso-lateral connective with it's caudal perikaya (*cp*). Myofibrils diverge from the orthogonal muscle grid as lobe formation commences, a circular muscle loops over the oral hood (arrowhead). **(C) **Left lateral view 10 hours before hatching showing areas of double muscle fibers (arrowhead), flattened circular muscles (double arrowheads) and circular nerves of the dorsal plexus (asterisks) following the anterior primary circular muscles (apcm). Scale bars 100 μm.

From 170 hpo (87%), muscles began to deviate from the orthogonal grid. On the ventral side circular muscles arched from one developing ventrolateral lobe up over the oral hood and down to the opposite ventrolateral lobe (Fig. 9B). A series of concentric muscle rings formed around the pharynx (Fig. 9B), and parenchymal mouth dilator muscles developed connecting the anterior margin of the mouth to the apical complex and circular body wall muscles. From 176 hpo (90%) onwards the roots of the apical tuft cilium was visible above the apical complex (not shown). Serotonin expression was detected in the pharyngeal nerve ring (Fig. 10D), which forms in close association with the pharyngeal circular muscles. Bilaterally symmetric pharyngeal perikarya (up to 4 in number) develop at this stage. From the two lower perikarya on the nerve ring two axons extend caudally and converge at a ventro-caudal perikaryon, these nerves then run adjacent to the primary longitudinal muscles and join the dorso-lateral connective at the caudal cilium.

Between 180-196 hpo (92-100% d.t.) many of the spiral muscle fibres of the apical complex are transformed into a more or less circular muscle system. Secondary longitudinal and circular muscles were observed to line up with the primary muscle fibres of the orthogonal muscle grid to give areas of double fibers (Fig. 9C). Many circular muscles formed thin flattened laminar sheets from two fibers (Fig. 9C). The number of acTub expressing intra-epidermal cells increased and the commissural cell bodies migrated further from the epidermis and closer together. The dorso-lateral connectives ran adjacent to the prominent double-stranded lateral longitudinal muscles that form part of the lateral lobes and weave either side of the circular body wall muscles (Fig. 9C). Below the lateral lobe this nerve crosses the body wall muscles and proceeds in the epidermis to the caudal tuft. A nerve plexus extends dorsally from the dorso-lateral nerve cord with two circular nerves running external to the circular muscles of the oral hood and dorsal lobe (Fig. 9C).

## Discussion

In this study, confocal laser scanning microscopy combined with fluorescent staining and immunohistochemistry has enabled the detailed description of polyclad cleavage, gastrulation, ciliation, myo- and neurogenesis, and larval myo- and neuroanatomy. This is the first study to detail the development and topology of the larval nervous system using antibodies against neurotransmittors, and to demonstrate the position of neural elements relative to musculature. It is now possible to compare the results found in *Maritigrella crozieri *amongst other polyclads and platyhelminths. The anatomy and development of other spiralian larvae have received considerable attention using these techniques (see [27] and references therein). The addition of embryological and larval data from a polyclad widens the taxon sampling within the Spiralia, giving a more comprehensive comparative framework within which to discuss the evolution of larval anatomies.

Polyclads are the only free-living platyhelminths where embryos can be reared *in vitro *outside of their impermeable egg capsule. As such they are an important taxon with which to address platyhelminth development and investigate the spiral pattern of early cleavage. In *Maritigrella crozieri *cleavage is of equal quartet spiral type and identification of the D quadrant occurs late, at the onset of epiboly. There appears to be no difference in blastomere size at the two or four-cell stage. In polyclads the macromeres are much larger than the first to third quartet micromeres. However, the sixth cleavage cycle gives rise to significantly larger micromeres than macromeres at the 4^th ^quartet stage. This may be a derived feature of polyclads, although verification in other archoophoran Rhabditophora such as macrostomids has not been possible as cleavage patterns beyond the third division are obscured by yolk [28]. However, an observation of a similar pattern has been recorded in the neoophoran Lecithoepitheliates [29].

Anti-tubulin immunohistochemistry on *Maritigrella crozieri *embryos showed that the ciliary band is the first ciliary feature to appear, well before the apical and caudal cilia. The ciliary band forms post-gastrulation as a complete continuous band around the stomodaeum, which then migrates to the position of the developing larval lobes. Ruppert [22] describes the distribution of prototrochal cells in another Muller's larva as a circular pre-oral band interrupted by the eight arm-like outgrowths of the body wall. However, there is variation in cilial band morphology in 4 and 6-lobed polyclad larva. In *Imogine mcgrathi *one ciliary band forms a complete circle around the larva at the latitiude of the oral hood, while a second ciliary band encircles the two lateral, peri-oral lobes [21]. In *Pseudoceros canadensis*, the ciliary band consists of discontinuous ventral and dorsal loops and ciliated features on the suboral plate [23]. This variation could be related to feeding strategies. The larvae of *M. crozieri *are actively planktotrophic [26] and the mechanism for planktivory is assumed to be the use of the ciliary band for down-stream feeding. *I. mcgrathi *larvae have been reported to feed passively on microplankton [21]. There is no evidence on whether *P. canadensis *is planktotrophic, however, given the accessory ciliary features that it possesses [23] it is highly likely that these are involved in feeding. Tubulin staining also highlights the apical and caudal cilia, the cilia in the developing pharynx, gut lumen, and paired protonephridia. It has also been used to visualize axon fiber development [21] (discussed below). A collar of cilia at the junction of the stomodaeum and larval gut is present in *M. crozieri *as in other polyclad larvae investigated by Ruppert [22].

### Myogenesis and larval myoanatomy

Adult musculature of Platyhelminthes consists of body wall muscles made up of circular, longitudinal and diagonal myofibrils, and parenchymal muscles [30]. Compared to other Platyhelminthes, there is a paucity of data on the musculature of adult polyclads, with data for only three species: *Planocera gilchristi *[31], *Notoplana acticola *[32,33] and *Discocelides langi *[34]. However, there are ontogenetic data on muscle development for three species: *Melloplana ferruginea *[20], *Hoploplana inquilina *[19] and *Maritigrella crozieri *[[20], this study]. The later two are indirect developers.

Comparisons between *Maritigrella crozieri *and *Hoploplana inquilina *highlight the similarities in muscle development and topology between six and eight-lobed larvae from two different species in different sub-orders (Cotylea and Acotylea respectively). Similarities in myogenesis include: 1) the early formation of a primary muscle grid, composed of two longitudinal muscles, an anterior and a posterior circular muscle. 2) An initial period of circular muscle development followed by longitudinal muscle growth. 3) Similarities in mouth retractor muscle position and number, and rings of concentric circular muscles around the mouth. The radial pharynx muscles described in *H. inquilina *probably equate to the parenchymal mouth dilator muscles described in *M. crozieri*. 4) Similar chronology and timing of larval lobe development, with the oral hood and ventro-lateral lobe anlages visible at 92% development time in *H. inquilina *and by 89% in *M. crozieri*.

However, there are several notable differences in muscles development and topology between *M. crozieri *and *H. inquilina*. The first primary longitudinal muscles of *H. inquilina *were located in lateral positions compared to dorsal positions in *M. crozieri*. Two transverse muscles extend over the apex of *H. inquilina *embryos at 92% development time and these were not observed in *M. crozieri *embryos. Pharyngeal longitudinal muscles described in *H. inquilina *were not observed in *M. crozieri*. A prominent diagonal muscle runs around the dorsal side of the embryo in *H. inquilina*. This is not in *M. crozieri *embryos, though one diagonal muscle was counted in two-day old larvae. This muscle becomes thicker over the following 8 days, by which time further diagonal muscles have formed. This suggests that in indirect developing polyclads diagonal muscles are added to the body wall musculature during the planktonic period. The observed acceleration of diagonal muscle development in *H. inquilina *relative to *M. crozieri *correlates with length of time in the plankton. *H. inquilina *larvae have been observed to settle at 16 days post-hatching [11] while *M. crozieri *larvae settle after approximately 3.5 weeks [35].

At hatching, the direct developing *Melloplana ferruginea *have approximately 10 pairs of diagonal body wall muscles on the dorsal side and 5 on the ventral side, many parenchymal dorso-ventral muscles, and a more elaborate musculature of the pharynx [20] compared to *Maritigrella crozieri *larvae. *M. crozieri *larvae develop diagonal muscles throughout the pelagic period and it is predicted that dorso-ventral muscles develop in later larval stages, thereby giving *M. crozieri *pelagic forms a similar body wall musculature to that of direct developing hatchlings at time of settlement. As such a comparable sequence of body wall musculature differentiation is seen between the different developmental modes; with the initial establishment of the orthogonal grid of circular and longitudinal muscles, followed by the formation of diagonal and dorso-ventral muscles. Comparisons between *M. crozieri, H. inquilina *and *M. ferruginea *reveal heterochrony in the development of two muscle types that may correlate with the different life history strategies. Both diagonal body wall muscles and complex pharyngeal muscles develop earlier in species that spend no (*Melloplana ferruginea*) to little (*Hoploplana inquilina*) time in the water column compared to species that spend longer periods feeding in the plankton (*Maritigrella crozieri*).

Features of the larval anatomy may be retained in the adult body plan as metamorphosis consists of a series of gradual changes involving the dorso-ventral flattening of the body and the reabsorption of the larval lobes [22,36]. This is quite unlike the catastrophic movements that effect metamorphosis in some spiralian larvae (e.g. nemerteans [37] and bryozoans [38]). Data from late stage polyclad larvae of many different species collected from the plankton show that growth of diagonal body wall muscles and dorsoventral parenchymal muscles dorso-ventrally flatten the larva whilst it is still actively swimming in the water column (Rawlinson, unpubl data). It is highly likely that the body wall musculature that develops in the *Maritigrella crozieri *larva persists through to adulthood and this is likely to be a common feature of polyclad larval development. Development of the musculature associated with a ventral sucker has been observed in late-stage larvae of unidentified species (presumably belonging to the sub-order Cotylea) (additional file 1 figure S1d), though whether this occurs in *M. crozieri *is unknown. Post-settlement myogenesis must continue in order to develop the high density of circular, longitudinal and diagonal body wall muscles and the complex muscle systems associated with the powerful tubular pharynx, the tentacles and reproductive structures of the adult worm.

"Intermediate-type" is the term used by Wanninger [27] to differentiate the mode of development of circular muscles in indirect developing polyclads from that in other worm-like spiralians. Based on myogenetic descriptions from *Hoploplana inquilina *[19] and *Pseudoceros canadensis *(Semmler and Wanninger, unpubl as cited in [27]), he summarises early myogenesis as the formation of pairs of secondary circular muscles in between the original two primary circular founder muscles, which develop synchronously at opposite ends of the embryo. Fission or duplication of existing muscle fibres is considered as the driving force in ring muscle growth in *P. canadensis *(Semmler and Wanninger, unpubl data as cited in [27]). This pattern of circular muscle distribution is also found in *Maritigrella crozieri*. However, the mechanisms that lead to the regular distribution of these circular muscles are not clear. Early myogenesis in *M. crozieri *shows myofibrils developing from the apical complex and primary circular muscles by means of fission (as seen in fibres with forked ends), and duplication (as seen in fibres lying in very close proximity to each other). Furthermore, circular muscles were observed to branch off from the primary longitudinal muscles.

While fission and duplication of pioneer muscle fibers appear to be the mode of myogenesis in *Maritigrella crozieri*, it has been suggested that nerve cords play a central guiding function during the development of circular muscles in the closely related rhabditophoran *Macrostomum hystricinum marinum *[39]. In *M. hystricinum marinum *migration and differentiation of pre-myocytes of circular muscles appear to be spatially restricted to the nerve cord. The cell bodies of these muscle fibers stay close to the main longitudinal nerve cords sending out the contractile part of their cells in a dorsal and ventral direction. In F-actin and serotonin labelled *M. crozieri *embryos, circular muscles form before the dorso-ventral and dorso-lateral connectives are visible (Fig. 8B and 8C) and early myofibrils are not spatially restricted to the developing dorso-lateral connective but are evenly distributed around the circumference of the embryo. Communication between these cell types is highly probable, as later in development the prominent lateral longitudinal muscles in *M. crozieri *do form in alignment with the already developed dorso-lateral connectives (Fig. 9C). Furthermore, there is close association between the cell types, particularly around the mouth and pharynx.

### Neurogenesis and larval neuroanatomy

Polyclad larval neuroanatomy has been described at the ultrastructural level in unknown species of Göttes and Müllers larvae [22] and in detail in *Pseudoceros canadensis *[23,24,40]. A cursory examination of the serotonergic nervous system has been carried out in *Stylostomum sanjuana *[41]. Development of the larval nervous system in *Imogine mcgrathi *was demonstrated at the ultrastructural level and using an antibody against acetylated tubulin [21].

An apical or frontal organ has been identified by ultrastructural studies in polyclad larvae [22-24] and a glandulo-sensory function has been suggested [22]. According to these studies, the apical organ consists of a cluster of monociliated sensory cells surrounded by a circle of gland cell necks supported peripherally by microtubules. The gland cell necks pass through the basement membrane extending posteriorly, dorsal and ventral to the neuropile to secretory cell bodies above, below and behind the brain [22,24]. The bases of the monociliated sensory cells were located on top of the brain (called apical cells, Lacalli [24]), but no direct connection to the brain was observed, Lacalli [24] therefore determined that these apical cells were probably not nerve cells.

Immunohistochemical studies [[21], this study] show immunoreactivity in the form of an apical plate. 5HT and FMRFamide are expressed in a ring around the apical cilia and these expression patterns may be located in the monociliated sensory cells described by Lacalli [23,24]. If the apical organ described in *Pseudoceros canadensis *is in the same position in *Maritigrella crozieri *then there appears to be little 5HT or FMRFamide immunoreactivity in the organ. It is possible that some of the acetylated tubulin staining visible intraepidermally under the apical plate is associated with the microtubules that give peripheral support to the gland cell necks (Fig. 7A). Furthermore the lack of immunoreactivity in the apical cells described by Lacalli [24], may confirm his suspicions that these cells lying on top of the brain (that give rise to numerous microvilli and to one cilium) are not nerve cells, although this requires further analysis, as many additional neuropeptides and neurotransmittors are found in larval nervous systems [42,43]. These findings may suggest that the frontal/apical organ (*sensu *Ruppert and Lacalli) may be more glandular than sensory, but that it is surrounded by neural activity in the neuropile and apical plate. Hay-Schmidt [41] described two serotonergic cells in the 'apical ganglion' of *Stylostomum sanjuana *larvae. These serotonin-expressing cells are equivalent to the commissural cell bodies described here in *M. crozieri *and the lateral cell masses described in *P. canadensis *[23]. They are not homologous to the serotonergic epidermal cells found in the apical organ of some other spiralian larvae (see below). Comparative studies on direct developing polyclad species would determine whether these species have a similar apical sensory plate or whether it is a unique character of the pelagic life history stage. Cilia at apical and caudal ends, with a presumed sensory function, have been identified in direct developing hatchlings [44].

Acetylated tubulin-expressing intra-epidermal cells at the anterior end of larvae have been recognized in *Imogine mcgrathi *('apical gland cells' in [21]) and *Maritigrella crozieri *(this study). In *Imogine mcgrathi *14-17 of these cells were found in later stage embryos, similarly in *Maritigrella crozieri *larvae, 2-8 acetylated tubulin-expressing cells formed bilaterally symmetric clusters in the dorsal epidermis at the latitude of the cerebral eyes. It is possible that these acTub expressing cells are homologous to the intra-epithelial multiciliated cells buried entirely within the epithelium described in *Pseudoceros canadensis *by Lacalli [24]. He concludes that due to their multiciliated organization that there should be some involvement with sensory reception and the nervous system. Findings in *M. crozieri *corroborate this hypothesis as fine connections were observed leading from these cells to the neuropile (Fig. 5A, F). Furthermore their strong acTub immunoreactivity could be consistent with dense ciliation. There are two differences observed between the acTub reactive intra-epidermal cells in *Imogine mcgrathi *and *Maritigrella crozieri*. Firstly, the processes leading from these cells are fine (much thinner than intra-epithelial nerves) in *M. crozieri *but thick and swollen in *I. mcgrathi*. Secondly, the processes from these cells extend to the brain in *M. crozieri*, whereas in *I. mcgrathi *they end at the apical plate. These differences may be due to inter-specific variation, or differences between larval types (Müller's vs Götte's). It is possible that the intra-epidermal acTub reactive cells observed in *M. crozieri *and *I. mcgrathi *and the intra-epithelial multiciliated cells in *P. canadensis *are ciliary photoreceptors and that these may persist through ontogeny to the adult body plan. In adult macrostomid species and other rhabditophorans putative light-perceiving organs are common, and these consist of pericerebral aggregations of cells with an internal cavity into which axonemata of modified cilia project [45]. Ciliated cells and 'sensory cups' (showing acTub immunoreactivity) associated with apical sensory organs have been described in the larvae of the polychaete *Platynereis dumerilii *[46] and the gastropod *Nerita melanotragus *[47]. In *P. dumerilii *these cells have been shown to express opsins [46], suggesting that they are ciliary photoreceptors and might function in non-directional photoresponse such as the control of photoperiodic behaviour. Indeed extraocular photoreceptors are found in many marine larvae [48]. To determine whether these intra-epidermal cells in *M. crozieri *are ciliary photoreceptors, the expression of opsins could be investigated and ablation experiments used to demonstrate their role, plus future developmental studies on later stage larvae and juveniles are needed to determine whether these cells persist in the adult body plan.

The anatomy of the larval neuropile is generally consistent between *Pseudoceros canadensis*, *Imogine mcgrathi *and *Maritigrella crozieri*. It consists of a spherical tangle of cell processes called the apical plexus (or median cluster in *I. mcgrathi *[21]), which lies anterio-dorsal to the orderly array of neurites that form the cerebral commissure. The neurites associated with the receptor cells of the cerebral eyes are distinctive for their large diameter in *P. canadensis *and rather than entering the brain directly, these pass down the side of the brain on either side to the ends of the commissure. The same pattern is seen in the ocellar nerves of *M. crozieri*. The lateral cell masses described in *P. canadensis *[23] probably correspond to the commissural cell bodies in *M. crozieri*.

Lacalli's [23,24] work on the neuroanatomy of the Muller's larva of *Pseudoceros canadensis *revealed the organization of the central and peripheral nervous systems. He described the central nervous system as comprising of a brain, apical organ and four nerve cords, all of which lie beneath the basement membrane. The peripheral nervous system is intra-epithelial and lies entirely outside the basement membrane consisting of nerves in association with the ciliary band. The only direct contact between the central and peripheral systems occurs at the ends of the dorsolateral cords, where a few neurites cross the basement membrane to the adjacent ciliary nerves [23]. A similar topology of nerves is seen in the larva of *Maritigrella crozieri *using immunohistochemical techniques, although some differences are recorded. In *M. crozieri *the distinction between a central and peripheral nervous system is not as distinct, as nerves appear to cross the basement membrane in at least two places. First, contrary to the findings in *P. canadensis *where the dorso-lateral connectives terminate at the ciliary band [24], the dorso-lateral connectives in *M. crozieri *extend to the lateral lobes, where they become external to the body wall musculature and continue in the sub-oral plate to the caudal tuft where they meet their co-lateral pair. Secondly, nerves associated with the pharyngeal nervous system may cross the basement membrane, especially FMRFamide expressing nerves. These nerves connect to the intra-epidermal caudal tuft perikaryon via the medial nerve and to the supra-oral nerve ring, while also connecting, sub-epidermally, to the dorso-lateral nerves and brain. Several other notable differences are observed in neuroanatomy of *Pseudoceros canadensis *and *Maritigrella crozieri *larvae; 1) the ventro-lateral cords terminate at the sides of the oral hood in *P. canadensis*, whereas in *M. crozieri *the two ventral connectives bifurcate, with one branch meeting it's co-lateral pair at the distal portion of the oral hood and the second branch extending posteriorly. 2) In *P. canadensis *suboral plate innervation consists of three nerve cells below the rejectory cell (a ciliated cell that functions as a food rejection system)[41]. However, in *M. crozieri *there is considerable sub-oral plate innervation as intra-epidermal innervation surrounding the caudal tuft and leading to the pharyngeal nerve ring.

In *Pseudoceros canadensis *Lacalli [23] found that most neural elements outside of the basement membrane belong to a separate intraepithelial system of nerves associated with the ciliary band. These ciliary nerves ran beneath the band along most of its length, the largest being those in the ventrolateral lobes where the sensory cells are the most numerous. A large nerve passed above the mouth to connect the ciliary nerves of the ventrolateral lobes. In *Maritigrella crozieri *5HT and FMRFamide immunoreactive cells beneath the epidermal cilia indicate a sub-cilial nerve plexus, these increase in density beneath the ciliary band, forming a prominent network following its entire length. No distinct nerve tracts were identified with the three neural markers used. There appeared to be a second nerve plexus beneath the sub-cilial plexus but above the body wall musculature, which was situated between and in contact with both the ciliary nerve band and the body wall musculature. The differences in neural anatomy described between *M. crozieri *and *P. canadensis *may be variation between species, artifacts of different types of analysis (immunohistochemistry vs electron microscopy) or ontogenetic differences whereby the smaller (150 μm anterior-posterior axis), less elaborate (6 lobes) hatchling of *P. canadensis *is slightly less developed.

A supra-oral ring of FMRFamide expressing cells was found in *Maritigrella crozieri *larvae (Fig. 4B). This has not been described from other Müller's larvae but Ruppert [22] observed an anastomosis of intraepithelial neurons between the two ventral clusters of neurons just anterior to the mouth in a Götte's larva. Perhaps this zone of neural activity is associated with a food groove as found in other spiralian larvae, where heavily innervated cilia convey particles towards the mouth or control mucous secretion.

A comparison of the larval serotonergic nervous system is possible between the 8-lobed larvae of two closely related euryleptids; *Stylostomum sanjuana *[41] and *Maritigrella crozieri *(this study). Hay-Schmidt [41] describes an apical ganglion housing a pair of serotonergic cell bodies from which an axon extends laterally towards the posterior end where they join a ring of serotonergic processes coming from six serotonergic cell bodies (one for each lateral lobe). Projections from the serotonergic neurons enter the ciliary band and the serotonergic larval nervous system initially stops at the prototroch. However, the serotonergic nervous system in *S. sanjuana *looks remarkably similar to that of *M. crozieri *and on close examination of the images I would disagree with Hay-Schmidt's interpretation that the six serotonergic cell bodies represent one for each lateral lobe and that the nervous system does not extend caudally beyond the ciliary band. The six serotonergic cell bodies he has indicated (his Fig 1A) look equivalent to those of the dorso-lateral connective in *M. crozieri*, which are found in the sub-oral plate.

From this study it is evident that early neurogenesis in *M. crozieri *starts with neurotransmittor (5HT and FMRFamide) immunoreactivity in the epidermis with the establishment of brain and apical plate primordia, and expression in caudal perikarya. The 5HT and FMRFamide expressing dorso-lateral and ventro-lateral connectives migrate posteriorly. The dorso-lateral connective connects the cerebral commisure to the caudal perikarya and the thinner, less prominent ventro-lateral connective bifurcates with one extension following the oral hood and the second following the inner margin of the future ventro-lateral lobes. Neurogenesis has only been studied in one other polyclad, *Imogine mcgrathi*, this was carried out using immunohistochemistry against acTub [21]. *I. mcgrathi *shows asymmetric development of brain - with the neuropile being thicker and extending further on the right side, with the right cerebral eye developing first. At the two eye stage in *I. mcgrathi *(their stage 7) a small dorso-medial cluster of neurons forms in the brain that sends branched processes toward the apical tuft. This is interpreted as a forerunner of the apical plexus. Antibody staining of *I. mcgrathi *was not achieved after this stage, however, this was possible in *Maritigrella crozieri *and it can be confirmed that this medial cluster is most likely the anlage of the apical plexus ('tangle') of the larval neuropile.

Lacalli [24] considered that the adult and larval orthogons cannot be considered equivalent or directly comparable without further information on how the former develops from the later. It is unlikely that the adult nervous system develops entirely separately from the larval nervous system due to the gradual nature of polyclad metamorphosis [22,36]. The neural anatomy of the benthic stage *Maritigrella crozieri *is unknown. However, from the acotylean, *Notoplana acticola*, Koopowitz [49] described the adult nervous system consisting of two nerve networks that radiate outwards from an anterior brain: 1) a ventral network of coarse nerves with a meshwork of finer fibres between the large nerves, 2) a dorsal network of fine fibres. It is hypothesised that the transition in *M. crozieri *from pelagic multi-lobed larvae to dorso-ventrally flattened benthic adult may include the following rearrangement to the nervous system topology: the prominent dorso-lateral connectives become the main ventral nerve cords in the flattened adult; the dorsal ring nerves become commissural nerves connecting the lateral nerves dorsally and forming part of the dorsal network; the 5HT expressing ventral perikaryon on the nerve cords that extend between the caudal tuft and pharyngeal nerve ring become associated with the ventral sucker.

If indeed the rearrangement of muscles and nerves involved with settlement to the benthos is gradual and slight, then perhaps the polyclad gastrula embarks on development towards a juvenile, but the early stages of this process co-exist with transient larval structures (e.g. lobes and ciliary band) to allow a temporary pelagic life and the ability to capture food. A further study on late stage larval development and settlement of *Maritigrella crozieri *would allow the morphogenetic change involved in settlement to be assessed. Furthermore, a working phylogeny of polyclads is needed to determine the polarity of change for life-history evolution within this clade. If an indirect life history was plesiomorphic then one might ask the role of the larval muscles and nerves in the development of the juvenile/adult body plan. If, however, the intercalation of pelagic stages occurred many times independently then one might consider how elements of the juvenile/adult musculature and nervous system have been co-opted or embellished to serve in mechanic and sensory roles in planktonic larvae.

### Spiralian larval ciliary bands and apical sensory organs

Neither spiral cleavage nor planktonic life stages are found universally amongst the Platyhelminthes or the Spiralia. Yet, it has been proposed that the trochophore larva represents the ancestral feeding larva of the Spiralia and possibly of the protostomes [50-53]. The name trochophore has been used in a fairly wide sense and has been redefined by Rouse [54] to include larval forms that have a prototroch originating from trochoblasts, an apical plate (and usually an apical tuft) and a pair of protonephridia. According to Rouse [54] the taxa that have a trochophore are the Annelida, Echiura, Entoprocta, Mollusca, and Sipuncula. Nielsen [13] added the Nemerteans and Platyhelminthes to this list. In accordance with Rouse's [54] definition, the polyclad larva could come under the term trochophore, and there is a long history defending a phylogenetic link between polyclad and trochophore larvae [10,13,22,53,55-58]. However, Elher's [59] platyhelminth phylogeny and recent molecular studies [[4-6], see [16]] have established a new framework for interpreting the distribution of larval forms within Platyhelminthes and the broader Spiralia. As polyclads are the only free-living Platyhelminth lineage to have planktonic larval forms, the likelihood of planktonic larvae being a primitive feature of this clade is low. Indeed, the primitive life history condition for polyclads remains unclear, due to the patchy distribution of larval characters within the group. Furthermore, the platyhelminths, themselves, are nested within a clade whose members do not universally exhibit planktonic larval stages. As such, it seems unparsimonious to suggest that the ciliary band and apical plate of polyclad larvae are homologous with those of other spiralian larvae. Nevertheless arguments of homology of larval traits have arisen due to morphological similarities in larval features, and from assumptions that the convergent evolution of these features is highly unlikely. The developmental data presented here from *Martigrella crozieri *enables a more detailed comparison of the ciliation, myo- and neurogenesis associated with the ciliary band and apical organ amongst spiralian larvae.

The prototroch is defined as a ring of (usually compound) cilia on multiciliate cells that is derived from trochoblasts, which exhibit a constant cell lineage and pattern of organisation [54,56,60,61]. In trochophore larvae, the prototroch is a pre-oral band of ciliary cells that function as the principal locomotory cilial band and as a motor generating a feeding current towards the mouth. In *Maritigrella crozieri *larvae the pre-oral ciliary band serves the same functions and homology of the polyclad ciliary band with the prototroch of other spiralian larvae has been proposed based on shared spiral cleavage [10], positional similarities [22], and the position of the cilial band cells along the borderline of first and second quartet micromere derivatives [[13] based on the interpretation of cell lineage data of [62]]. However, convergent evolution is suggested by Ax [1] and Jenner [63] based on the phylogenetic distribution of larval forms and characters. Rouse [54] suggests that a prototroch is absent in Platyhelminthes, based on the statement by van den Biggelaar et al. [64,65] that polyclad larvae are uniformly ciliated and do not posses a discrete ciliary band.

Data from *Maritigrella crozieri *reveal a number of similarities and differences between these ciliary bands and the associated muscles and nerves. Contrary to the findings of van den Biggelaar et al. [64,65] a single pre-oral band of cells with long cilia is present in *M. crozieri *(and in all other polyclad larvae examined by the author, unpub data). It develops following gastrulation and ciliation of the epidermis, and prior to the development of the apical or caudal cilia. The prototroch is also the first ciliary feature to appear in the trochophore larvae of some polychaetes [66,67] and mollusks [60]. There are two rows of cilia on the peri-oral lobes in *M. crozieri *ie. the oral hood and both ventro-lateral lobes. Two rows of cilia are also present in the prototroch of the trochophore of the polychaete *Pomatoceros lamarckii *[66].

Differences between the musculature and innervation of the ciliary band of *M. crozieri *larvae and the prototrochs of various spiralian larvae are noted. *M. crozieri *lacks a distinct prototroch muscle directly beneath the ciliated prototrochal cells. Prototroch muscle rings of this nature are found in some mollusk and annelid larvae but are lost at metamorphosis [17,66,68]. These muscle rings are lacking in some taxa with a very short larval phase such as the Scaphopod, *Antalis entalis *(metamorphic competence reached 94 hours postfertilisation)[69] and the Sipuncula, *Phascolion strombus *(metamorphic competence 72 hpf)[70]. In *M. crozieri *the ciliary band cells form a convoluted continuous band around the eight larval lobes, multiple longitudinal and circular body wall muscles are involved in the movement and orientation of the band during the comparatively long planktonic phase. As these muscles are part of the orthogonal grid of body wall musculature, it seems unlikely that they are truly larval muscles and probably become part of the juvenile musculature.

The serotonin immunoreactivity, in the form of a dense nerve net between the ciliated cells of the ciliary band and the muscles of the lobes of *M. crozieri*, bares more resemblance to the extensive nerve net in polyplacophoran [71] and ectoproct larvae [72] than to the distinct serotonin-labelled axons of the prototroch nerve rings of the larvae of some polychaetes [66], bivalves [73] and gastropod mollusks [74]. In early *Platynereis dumerilii *trochophores, ciliary band beating and phototactic movement is under the control of larval photoreceptors that are in direct contact with the prototroch; the two larval photoreceptors work as sensory-motor neurons [43]. In *M. crozieri *larvae there is no direct coupling of light sensing and ciliary motor control as the eyespots are not located near the ciliary band. Instead neurons from the eyes are centralized through the brain and integrated neuronal processes probably coordinate ciliary band beating and the activity of longitudinal, circular and diagonal muscles to steer the larvae. Sensory and motor nerves may already have differentiated before hatching in *M. crozieri*. Future investigations at the molecular level will help determine whether there are similar developmental mechanisms involved in ciliary band morphogenesis between larvae of different taxa, and the early appearance of the ciliary band in polyclad embryos may serve as a reference for embryonic gene expression patterns.

Many marine protostome larvae have an apical cluster of neurons positioned below an apical cilium or patch of cilia. Neurons within this apical ganglion are among the first cells to express a neuronal phenotype during embryogenesis [75-78], and it has been suggested that these cells induce the development of the cerebral system [79,80]. During the pelagic stage of the life-history it is generally considered that this apical ganglion functions in a sensorimotor capacity to transduce environmental stimuli into motor signals that modulate muscular and ciliary activities of the larva [74], eventually triggering larval settlement and metamorphosis [81,82].

It is becoming evident that many neurotransmittors are expressed in these apical neurons [43,83]. However, due to the commonly available antibody to serotonin and its conservation across the Metazoa [41], this neurotransmittor and its spatial and temporal distribution in embryonic and larval nerves has received most attention to date. Apical clusters of serotonin expressing larval neurons have been called a variety of names including 'apical sensory organ', 'cephalic sensory organ', apical disc', 'apical organ' and 'apical ganglion' and they have been described from species in the following spiralian clades: Mollusca [42,71,74-76,80,84,85], Annelida [66,86,87] Sipuncula [88], Phoronida [89,90], Bryozoa [91], Brachiopoda [83], Entoprocta [72].

There is great diversity in shape, number and immunoreactivity of the apical ganglion neurons amongst spiralian larvae, even within phyla, with apical ganglia missing all together in Echiura [92,93], the coronate larvae of a bryozoan [[38], although see [91]] and the choroid larva of Cycliophora [94]. Some of these serotonergic epithelial cells have neck-like apical projections giving them a flask-shape, as seen in many mollusk taxa [71,73,74,80,84], an entoproct [72]), and a polychaete [87]. However, other polychaetes [66,86], bryozoans [91] and phoronids [90] do not show such cell morphology. Wanninger [18,27] considers the plesiomorphic condition for spiralians to be a simple apical organ with about four serotonergic flask-shaped cells.

Phylogenetic homologies of the apical ganglion are difficult and need to be based on comparable developmental stages. Results on the ontogeny of the serotonergic nervous system in a *M. crozieri *show that, at hatching, a simple apical plate consists of a small ring of serotonin-positive epidermal cells under the ciliary ring surrounding the apical cilium (Fig. 4E). Therefore the intra-epidermal, flask-shaped serotonergic cells of the larval apical organ, considered plesiomorphic for the spiralians [18,27], are not found in the hatchling stage of *M. crozieri*. However, in order to ascertain that these apical organ cells are not found at different developmental stages a couple of further investigations are necessary. Firstly, in *M. crozieri *the embryonic transient ring of epidermal, serotonin-expressing cells between the cerebral eyes (Fig. 8D & 10C) shows similar positioning to the ring of flask-shaped cells of the larvae of a polyplacophoran [71] and an entoproct [72]. The close proximity of this ring of cells to the developing cerebral commissures and associated neuronal cell bodies would suggest a role in larval brain development, as has been proposed for Molluscs [80]. If this is the case, it could be hypothesized that the polyclad equivalent of the apical organ flask-shaped serotonergic cells is an embryonic feature and that the cerebral system is already established before the larval phase. Secondly, it is necessary to determine whether serotonergic flask-shaped cells develop in *M. crozieri *near time of settlement, as these cells are thought to function in chemo-sensory detection of metamorphic cues in the veliger of the gastropod *Phestilla sibogae *[95].

In the early *Maritigrella crozieri *larva the serotonin expressing cells in the apical plate and their connection to the neuropil are simple compared to the elaborate apical organs of many other spiralians, which may include apically derived structures as well as lateral ganglionic components. Whether the morphologically simple apical plate of polyclads is a primitive condition and complex apical organs have convergently evolved multiple times in separate lineages remains to be tested.

While this study has highlighted several gross morphological similarities and differences between the ciliary bands and apical organ across taxa, the current data does not allow the non-arbitrary distinction between parallelism, convergence and homology. A more robust phylogenetic analysis would allow better distinction between homology and parallelism, while more developmental data would help distinguish between convergence and parallelism [96]. Considering the patchy distribution of the ciliary band/prototroch and apical organ across the Spiralia, three scenarios of organ-level evolution can be considered.

1) Morphological homology - with the ciliary band and an apical organ being present in the pelagic stage of last common ancestor, with repetitive loss in multiple lineages explaining the unparsimonious distribution on the tree.

2) Morphological similarity because of parallelism. The developmental mechanisms to make the ciliary band and apical organ in the last common ancestor of Spiralia were recruited convergently in taxa with these features.

3) Complete convergence of ciliary bands and apical organs as similar adaptations to external conditions. Independently evolved features with different developmental mechanisms.

Scenario 1 is accepted by some [13,27] although, based on current spiralian and platyhelminthe phylogenies, the distribution of ciliary bands (and possibly apical organs) is not congruent with a hypothesis of homology. In order to differentiate between scenarios 2 and 3 developmental similarities can be investigated by searching for common gene expressions and by examining the regulation of these genes to assess whether the regulatory linkages are convergent or developmentally similar. This study has provided the anatomical framework upon which to test these scenarios.

## Conclusion

Compared to many spiralian taxa with biphasic lifecycles polyclad species with pelagic stages hatch with well-developed muscle and nervous systems. This study gives a further example of myogenesis and the myoanatomy of an indirect developing polyclad species and shows that the patterns of muscle development are similar between species with different modes of development and that differences in the timing of development of diagonal bodywall muscles and pharyngeal muscles correlate with length of pelagic stage and feeding strategies. The data also suggest that in *Maritigrella crozieri*, and other species with biphasic life cycles, that significant myogenesis continues over the pelagic stage, that these and muscles developed during embryogenesis are likely to persist in the adult bodyplan and that settlement to the benthos probably does not involve major rearrangement of bodywall muscles. These observations have bearings on the evolution of the biphasic life cycle and suggest that the intercalation of a pelagic stage may only require the development of a ciliary band and the lobes on which it sits.

Data on the development of the nervous system in *Maritigrella crozieri *show that at hatching the larva has developed sensory apparatus in the form of an apical cilium and apical plate, three pigmented photoreceptors, many putative ciliary photoreceptors (intra-epidermal cells) and a caudal cilium. These are integrated into a centralized nervous system through a simple neuropile and the paired nerve cords, which connect to the body wall musculature and ciliary band. This first study detailing the distribution of the neurotransmittors serotonin and FMRFamide, and demonstrating the position of neural elements relative to the musculature, provides the basis on which to examine the diversification of polyclad and spiralian larval body plans.

Comparisons of the ontogeny, musculature and neural system of the ciliary band in *Maritigrella crozieri *with the prototroch in other spiralian taxa reveal morphological similarities and differences, providing evidence for and against the homology of these structures. The many underlying morphological differences and the current distribution of ciliary bands/prototrochs across the platyhelminthe and spiralian phylogenies assumes that the covergent evolution of the polyclad ciliary band is most parsimonious.

## Methods

### Animal collection and culture

*Maritigrella crozieri *adults were collected from Zane Grey Creek on Long Key and from No Name Key in the Florida Keys, U.S.A. from August 2008 to February 2009. They were kept in seawater tanks and fed the ascidian *Ecteinascidia turbinata *(Herdman 1880). In order to carry out phalloidin staining, immunohistochemistry and nuclear staining on the developing embryos it was necessary to obtain eggs lacking the impermeable egg-shell membrane. This was achieved using the technique of Boyer [97], whereby the uteri of gravid adults were pierced with a sharpened tungsten needle to release mature, fertilized eggs. Naked embryos were raised in penicillin-streptomycin (100 μg/ml penicillin; 200 μg/ml streptomycin) Millipore filtered seawater in gelatin coated Petri dishes at room temperature (22°C), with daily water changes.

### Sampling and fixation

Embryos were collected at approximately 5 hours intervals. Embryos were fixed for 1 hour at room temperature in 4% formaldehyde in 0.1 M phosphate buffered saline (PBS), pH = 7.6. Embryos were rinsed in PBS for 1 h at room temperature. Embryos for phalloidin and nuclear staining were stored in PBS and 0.1% sodium azide at 4°C. Embryos for immunohistochemistry were dehydrated and stored in Methanol at -20°C.

### Phalloidin staining and immunohistochemistry

To visualize filamentous actin, embryos were washed 3 × 15 min with 0.1 M PBS, permeabilised for 1 h in PBS and 0.1% Triton x-100 (PBST), and incubated in Alexa 488 or 543 phalloidin 1:500 (Molecular Probes) for 1 h at room temperature. Embryos were washed in PBST for 1 h. Blastula and gastrula stage embryos were then counter-stained with the nuclear stain Sytox Green (Invitrogen)(0.1 uM Sytox Green for 30 min in the dark, rinsed for 1 h in PBST).

To visualize neurogenesis and development of cilia, three primary antibodies were used; anti-serotonin (immunostar) and anti-FMRFamide (immunostar), which label the cytoplasm of a subset of central nervous system neurons (cell body and axonal processes), and anti-acetylated tubulin (Sigma), which labels the stabilized microtubules of neurons and ciliated cells. From methanol, embryos were rinsed three times in PBS followed by 4 × 20 min washes in PBST and then placed in Image-iT™ FX Signal Enhancer (Molecular Probes) for 1 h to minimize non-specific staining. Specimens were rinsed in PBST for 1 h and incubated in primary antibodies (anti-rabbit FMRFamide diluted 1:500, anti-rabbit Serotonin diluted 1:500, or anti-mouse Acetylated Tubulin diluted 1:500) for 18 h at 4°C. All antibodies were diluted with PBST. Specimens were then rinsed 4 × 30 min in PBST and transferred to goat anti-rabbit Alexa-Fluor 488 or goat anti-mouse Alexa-Fluor 488 (Molecular Probes), as appropriate (diluted 1:200 in PBT). Specimens were incubated in secondary antibody for 18 h at 4°C and then rinsed in PBST for 12 h. Negative controls were obtained at each developmental stage by omitting the primary antibody and no fluorescence signal was detected.

### Microscopy and image processing

Immunostained embryos were dehydrated (30%, 50%, 70%, 95% and 100% isopropanol), cleared with Murray's reagent (2:1 benzyl benzoate: benzyl alcohol) and mounted in Permount (Fisher). Phalloidin stained embryos/larvae were mounted in Vectashield antifade mounting medium (Vector Laboratories, Burlingame, CA). 10 or more embryos per development stage were imaged using a Zeiss LSM 510 confocal laser scanning microscope. Digital images were assembled in Adobe Photoshop CS.

## Competing interests

The author declares that they have no competing interests.

## Supplementary Material

Additional file 1 **Figure S1 - CLSM micrographs showing musculature and serotonergic nervous system of polyclad pelagic stages**. (phalloidin - white, 5HT - magenta). **(A) **10 day post hatching *Maritigrella crozieri *larva, dorsal view, showing development of diagonal body wall muscles above the dorsal lobe and the dorsal diagonal arc (arrow heads). **(B) **2 day post hatching *M. crozieri *larvae showing two rows of cilia on the ciliary band of the peri-oral lobes (arrowheads) and one row on the lateral and dorso-lateral lobes (double arrowhead). **(C) **Right lateral view of 2 day post hatching *M. crozieri *larvae showing extensive synaptic serotonin expression between receptors of the ciliary band cells, axons and musculature of lobes (*cb *- ciliary band, *ap *- apical plate). **(D) **Unidentified 8-lobed polyclad larvae showing later-stage larval muscle development including development of ventral sucker (*vs*) muscles and further development of mouth (*m*) and pharyngeal (*ph*) muscles. Scale bars: A-C 50 μm, D 100 μm. Click here for file
