# Supplementary figures and images for: Embryonic and post-embryonic development of the polyclad flatworm Maritigrella crozieri; implications for the evolution of spiralian life history traits
Source: Front Zool. 2010 Apr 28;7:12. doi: 10.1186/1742-9994-7-12 (PMC2876153; doi:10.1186/1742-9994-7-12)

A

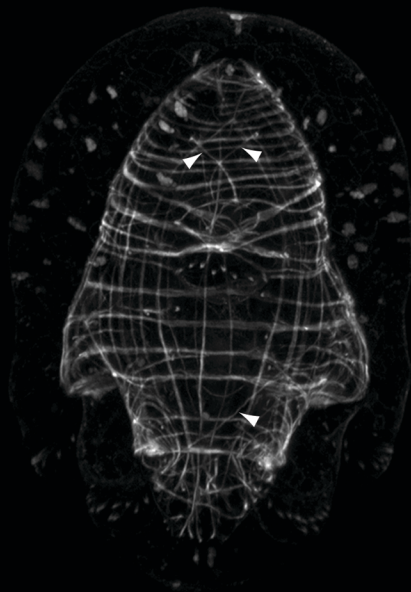

B

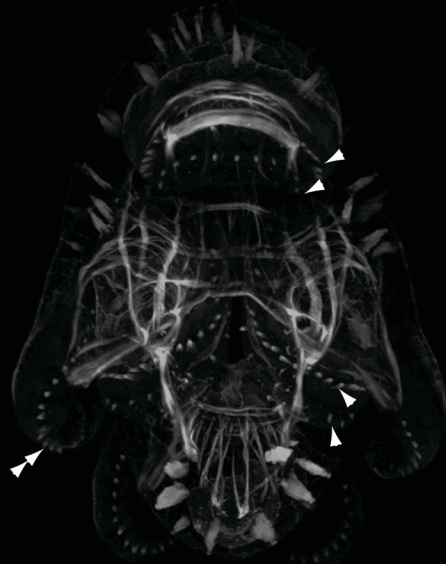

C

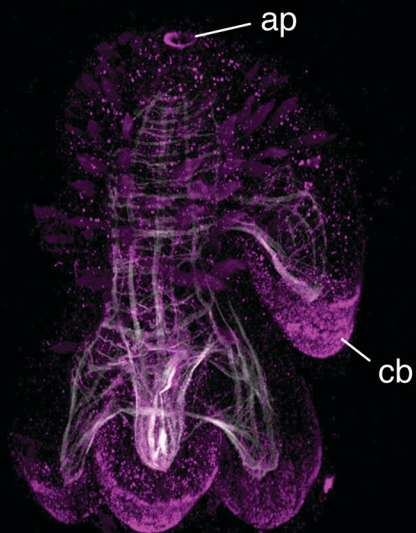

D

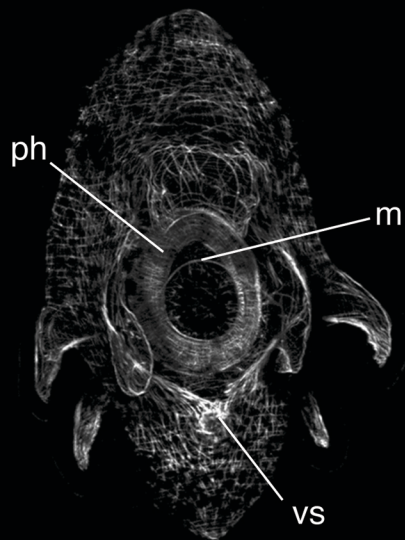

Supplement: Additional file 1 — Figure S1 - CLSM micrographs showing musculature and serotonergic nervous system of polyclad pelagic stages. (phalloidin - white, 5HT - magenta). (A) 10 day post hatching Maritigrella crozieri larva, dorsal view, showing development of diagonal body wall muscles above the dorsal lobe and the dorsal diagonal arc (arrow heads). (B) 2 day post hatching M. crozieri larvae showing two rows of cilia on the ciliary band of the peri-oral lobes (arrowheads) and one row on the lateral and dorso-lateral lobes (double arrowhead). (C) Right lateral view of 2 day post hatching M. crozieri larvae showing extensive synaptic serotonin expression between receptors of the ciliary band cells, axons and musculature of lobes (cb - ciliary band, ap - apical plate). (D) Unidentified 8-lobed polyclad larvae showing later-stage larval muscle development including development of ventral sucker (vs) muscles and further development of mouth (m) and pharyngeal (ph) muscles. Scale bars: A-C 50 μm, D 100 μm. [file 1742-9994-7-12-S1.PDF]
